# Comparative genomics of the class 4 histone deacetylase family indicates a complex evolutionary history

**DOI:** 10.1186/1741-7007-4-24

**Published:** 2006-08-02

**Authors:** Valérie Ledent, Michel Vervoort

**Affiliations:** 1 Belgian EMBnet Node, Laboratoire de Bioinformatique, Université Libre de Bruxelles, Institut de Biologie et de Médecine Moléculaires, Rue des Professeurs Jeener et Brachet 12, B-6041 Gosselies, Belgium; 2 Evolution et Développement des protostomiens, Centre de Génétique Moléculaire, UPR 2167 CNRS, 1, av. de la terrasse, 91198 Gif-sur-Yvette cedex, France; 3 UFR de Biologie et Sciences de la Nature, Université Paris 7, Denis Diderot, 2 place Jussieu, 75251 Paris cedex 05, France

## Abstract

**Background:**

Histone deacetylases are enzymes that modify core histones and play key roles in transcriptional regulation, chromatin assembly, DNA repair, and recombination in eukaryotes. Three types of related histone deacetylases (classes 1, 2, and 4) are widely found in eukaryotes, and structurally related proteins have also been found in some prokaryotes. Here we focus on the evolutionary history of the class 4 histone deacetylase family.

**Results:**

Through sequence similarity searches against sequenced genomes and expressed sequence tag data, we identified members of the class 4 histone deacetylase family in 45 eukaryotic and 37 eubacterial species representative of very distant evolutionary lineages. Multiple phylogenetic analyses indicate that the phylogeny of these proteins is, in many respects, at odds with the phylogeny of the species in which they are found. In addition, the eukaryotic members of the class 4 histone deacetylase family clearly display an anomalous phyletic distribution.

**Conclusion:**

The unexpected phylogenetic relationships within the class 4 histone deacetylase family and the anomalous phyletic distribution of these proteins within eukaryotes might be explained by two mechanisms: ancient gene duplication followed by differential gene losses and/or horizontal gene transfer. We discuss both possibilities in this report, and suggest that the evolutionary history of the class 4 histone deacetylase family may have been shaped by horizontal gene transfers.

## Background

In eukaryotes, DNA is packaged into chromatin structures, the basic unit of which is the nucleosome. Each nucleosome consists of about 148 bp of DNA tightly wrapped around a histone-protein octamer containing two copies each of H2A, H2B, H3, and H4 [1]. The packaging of DNA restricts its accessibility to proteins such as transcription factors, and therefore the transcriptional activation of many genes requires chromatin modifications such as reversible acetylation of the core histones [2]. The steady-state level of acetylation is controlled by the antagonistic activities of two types of enzymes: histone acetyltransferases and histone deacetylases (HDACs). HDACs thus play key roles in transcriptional regulation and also in other cell processes that are influenced by the acetylation state of core histones, such as chromatin assembly, DNA repair, and recombination [3,4].

HDACs have additional activities that are not directed at histones: many HDACs are partially found in the cytoplasm, and some have been shown to act on non-histone substrates, such as the cytoskeletal protein, tubulin, and the transcription factors p53 and YY1 [5-7]. Acetylation/deacetylation might thus be a widespread type of post-translational modification, acting in a manner similar to phosphorylation/dephosphorylation in the regulation of protein activity [8]. In addition, HDACs have recently attracted considerable attention because chemical inhibitors of HDACs induce growth arrest, differentiation, and/or apoptosis of cancer cells both *in vitro *and *in vivo*, and may thus represent a new class of anti-tumor agents [4].

Recent phylogenetic studies [9] classify the non-sirtuin HDACs into three families: the well-known class 1 (which includes the human HDACs 1, 2, 3, and 8), class 2 (including the human HDACs 5, 6, 7, 9, and 10), and an additional class defined by the recently identified human HDAC 11 [10]. This third class has been named class 4 to distinguish it from the unrelated NAD-dependent class 3, i.e. the sirtuin deacetylases related to the yeast Sir2 protein [9]. Orthologues of the eukaryotic HDACs are found in prokaryotes [9,11], and phylogenetic analyses indicate that most of them can confidently be assigned to one or another of the three classes distinguished among eukaryotic HDACs [9]. These prokaryotic proteins act biochemically on non-histone substrates and are usually labelled as 'acetoin utilization proteins' or 'acetylpolyamine amidohydrolases' with reference, respectively, to their involvement in the utilization of the carbon source acetoin or in the deacetylation of polyamines such as spermine [11]. It is known, however, that acetylpolyamine aminohydrolases share some important functional features with eukaryotic histone deacetylases, as both: (i) recognize an acetylated aminoalkyl group; (ii) catalyse the removal of the acetyl group by cleaving an amide bond; and (iii) increase the positive charge of the substrate [11].

In this study, we have identified, through similarity searches against sequenced genomes and EST data, a very large sampling of putative eukaryotic and prokaryotic proteins belonging to the class 4 HDAC family. In the remainder of this paper we call these 'class 4 HDACs' on the sole basis of their orthology to the characterized class 4 HDACs of metazoans, and irrespective of their actual functional specificities, which have not been characterized. By means of multiple phylogenetic analyses, we show that the class 4 HDACs display unexpected phylogenetic relationships, at odds with the phylogeny of the corresponding species. Some eukaryotic proteins appear more closely related to eubacterial proteins than to those of related eukaryotic species. We discuss the possibility that this anomalous phyletic distribution might be the consequence of multiple ancient horizontal gene transfers between prokaryotes and eukaryotes, or alternatively, the result of gene duplication and a high rate of differential gene loss.

## Results

### Derivation of a comprehensive set of class 4 HDACs

**Figure 1:**
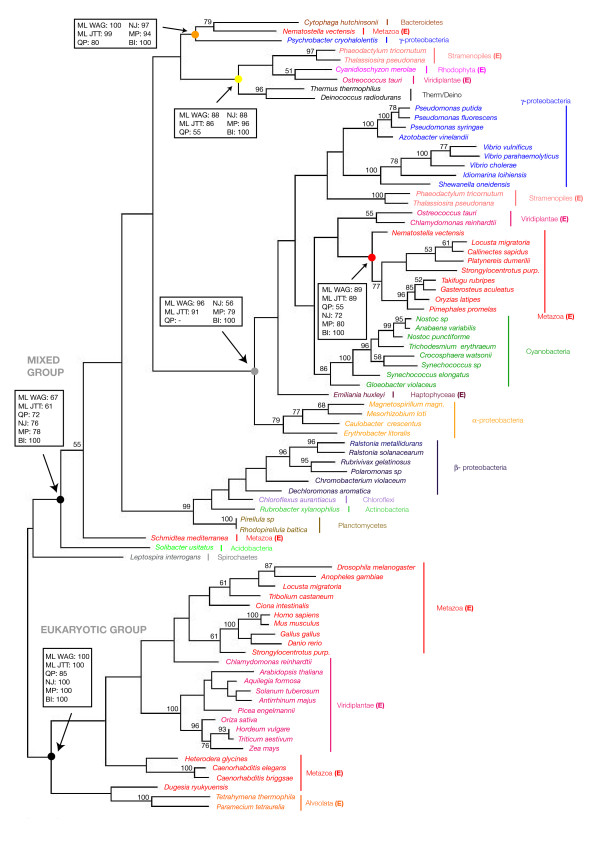
**Phylogenetic tree of the class 4 HDACs**. The represented tree is a maximum-likelihood (ML) tree produced with PHYML (using the WAG model of protein evolution). Rooting is arbitrary. Numbers above the internal branches are their bootstrap support values (100 bootstrap replicates). Only bootstrap support values >50% are shown. Other internal branches (with bootstrap support <50%) should be considered unreliable. In addition to the values shown for the other statistically supported internal branches (ML WAG), some key internal branches are labelled with additional statistical support values obtained by other methods of phylogenetic reconstruction or other models of evolution: bootstrap support in PHYML analysis with the JTT model of evolution (ML JTT; 100 bootstrap replicates), ML quartet puzzling support (QP; 25,000 puzzling steps), bootstrap support in neighbour-joining (NJ) analysis (BioNJ algorithm, 10,000 bootstrap replicates), bootstrap support in maximum-parsimony (MP) analysis (heuristic search; 500 bootstrap replicates), and posterior probabilities (Bayesian inference, BI). More details on the phylogenetic analyses can be found in the Methodssection and additional trees can be found in Additional Files 3, 4, 5. Eukaryotic lineages are marked by a bold "E". Colored circles highlight internal nodes defining monophyletic groups discussed in the main text. Black circles define two large groups: the "eukaryotic group" including proteins of species belonging to three of the main eukaryotic lineages, namely the opisthokonta (metazoa), plantae (viridiplantae), and chromalveolata (alveolata), and the "mixed group" comprising proteins of species belonging to various eubacterial and eukaryotic lineages. The grey circle denotes, inside the mixed group, a monophyletic group containing both eukaryotic and eubacterial sequences. The red circle indicates a monophyletic group comprising class 4 HDACs of nine animals; these HDACs show closer resemblance to eubacterial proteins than to those of other animals. The yellow circle defines a group comprising four HDACs of distantly related eukaryotic planctonic species. The orange circle denotes a group comprising an HDAC sequence of the cnidarian *Nematostella vectensis *and those of two unrelated eubacteria. The multiple alignment on which this tree is based is available as Additional File 8.

**Figure 2:**
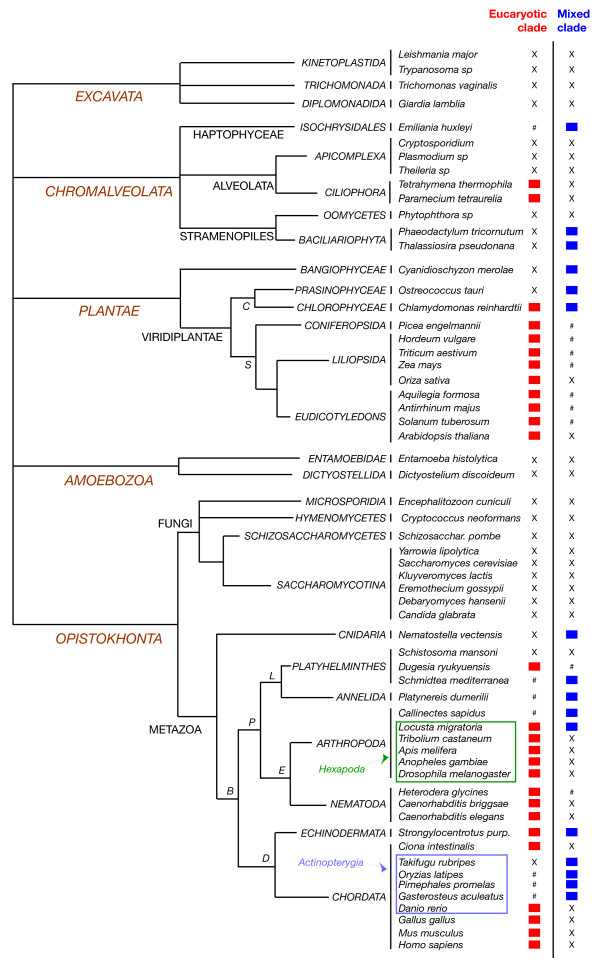
**Phyletic distribution of the eukaryotic class 4 HDACs**. Most of the species in which we sought class 4 HDACs are shown, together with our current view of their phylogenetic relationships [12]. The nomenclature used for the phylogenetic groups is according to Simpson and Roger [12] and the NCBI website. In each case in which the genome of a species has been fully sequenced, we indicate the presence or absence of a "eukaryotic group" or "mixed group" class 4 HDAC. The # symbol denotes species for which only EST data are available. In these cases, our failure to find a class 4 HDAC of one type of the other does not mean that none exists. In the case of the fungi, we searched against the genomic data of all species available on the NCBI website and failed to find a class 4 HDAC in any of them. '*Phytophthora sp*' are *Phytophthora sojae *+ *Phytophthora ramorum*; '*Plasmodium sp*' are *Plasmodium berghei *+ *Plasmodium chabaudi *+ *Plasmodium vivax *+ *Plasmodium yoelii yoelii*; '*Trypanosoma sp*' are *Trypanosma cruzi *+ *Trypanosma brucei*.

In a survey of animal HDACs, we found by Blast BLAST searches against the National Center for Biotechnology Information (NCBI) NR database that some animal class 4 HDACs are more similar to eubacterial HDACs than to those of other eukaryotes. In order to conduct a phylogenetic analysis of these sequences on the largest possible sampling of HDACs, we retrieved a large number of protein sequences of class 4 HDACs (HDAC11-related) by means of BLAST searches against the NCBI NR database, and also against various finished and unfinished genomes and expressed sequence tag (EST) data. To ascertain that the retrieved sequences were *bona fide *class 4 HDACs, we used both the "reciprocal best BLAST hit" criterion and phylogenetic analyses (as described under Methods and in Additional File 1). We identified class 4 HDACs in 45 eukaryotic and 37 eubacterial species representative of evolutionarily very distant lineages (Figures 1 and 2; Additional File 2). We failed to detect any class 4 HDAC in some archaea, fungi, and apicomplexa (*Plasmodium *and relatives). In the corresponding genomes, one or more HDAC sequences were found but they belong to HDAC class 1 and/or class 2 (not shown).

### Phylogenetic analyses of the class 4 HDACs

We performed a multiple alignment of the retrieved class 4 HDACs of 82 different species and used this alignment to construct phylogenetic trees. We then applied several different phylogenetic methods (as described in the legend of Figure 1 and under Methods) to reconstruct evolutionary relationships among the class 4 HDACs. We used both statistical support (bootstrap values, quartet puzzling support values, and posterior marginal probabilities) and congruence between the different phylogenetic methods as indicators of the reliability of the different internal branches of the tree. Figure 1 summarizes these results. The trees obtained by the different phylogenetic methods can be found in the Additional Files 3, 4, 5).

We found two large well-supported monophyletic groups (Figure 1; black circles). One group, which we named the 'eukaryotic group', includes only eukaryotic proteins of animals (metazoa), land plants and a green alga (viridiplantae), and ciliates (alveolata), i.e. of representatives of three of the main eukaryotic lineages [12] (opisthokonta, plantae, and chromalveolata, respectively). The other group, called the 'mixed group', includes proteins of representatives of various lineages, both eubacterial and of eukaryotic: animals (metazoa, opisthokonta), green algae (viridiplantae, plantae), a red alga (rhodophyta, plantae), diatoms (stramenopiles, chromalveolata), and a coccolithophore alga (haptophyceae, chromalveolata). This mixed group includes a well-supported monophyletic group (Figure 1; grey circle) comprising eukaryotic sequences and sequences from cyanobacteria and proteobacteria.

The phylogeny of the class 4 HDACs appears, in many respects, at odds with the phylogeny of the species in which these proteins are found. In the mixed group, we identified, for example, a monophyletic group of nine animal proteins showing closer resemblance to eubacterial proteins than to those of other animals (Figure 1; red circle). This group includes sequences belonging to representatives of several animal lineages: a cnidarian (*Nematostella vectensis*), two arthropods (*Callinectes sapidus*, a crustacean, and *Locusta migratoria*, an insect), an annelid (*Platynereis dumerilii*), an echinoderm (*Strongylocentrotus purpuratus*), and four vertebrates (the teleost fishes *Takifugu rubripes*, *Oryzias latipes*, *Gasterosteus aculeatus*, and *Pimephales promelas*). Strikingly, the class 4 HDAC found in these teleosts is only distantly related to that found in another teleost fish, the zebrafish *Danio rerio*, and is more closely related to eubacterial proteins (Figure 1). Similarly, one class 4 HDAC found in *Locusta migratoria *is closer to those found in eubacteria than to those of other insects (*Drosophila melanogaster*, *Anopheles gambiae*, *Apis melifera*, and *Tribolium castaneum*) and to the second class 4 HDAC of *Locusta migratoria*. In the mixed group, we also found class 4 HDACs in two green algae, *Chlamydomonas reinhardtii *and *Ostreococcus tauri *(Figure 1), appearing more similar to eubacterial class 4 HDACs than to those of other viridiplantae, such as *Arabidopsis thaliana*, *Oryza sativa*, or to the second class 4 HDAC of *Chlamydomonas reinhardtii*. We further noted the existence of a monophyletic group including proteins of very distant eukaryotic species (Figure 1, yellow circle): the diatoms *Thalassiosira pseudonana *and *Phaeodactylum tricornutum *(chromalveolata), the red alga *Cyanidioschyon merolae*, and the green alga *Ostreococcus tauri *(plantae). The green alga sequence is thus more closely related to those of the diatoms, which are evolutionarily quite distant, than to those of any other viridiplantae. Finally, we found a monophyletic group comprising the second HDAC sequence found in the genome of the cnidarian *Nematostella vectensis *and the sequences of two distantly related eubacteria, *Cytophaga hutchinsonii *(a Bacteroides species) and *Psychrobacter cryhalolentis *(a γ-proteobacterium) (Figure 1, orange circle).

There is thus a clear incongruence between the HDAC protein tree and the phylogenetic tree of the corresponding species. We then looked more closely at the distribution of class 4 HDACs in eukaryotes (Figure 2). Class 4 HDACs are found in three of the main lineages of eukaryotes (Chromalveolata, Plantae, and Opisthokonta) [12]. Inside each of these groups, some species possess proteins belonging to the eukaryotic group and others display proteins of the mixed group, with a few species possessing both types (Figure 2). The eukaryotic class 4 HDACs thus clearly display an anomalous phyletic distribution, given our current view of the phylogenetic tree of eukaryotes.

## Discussion

Two main mechanisms might account for the unexpected phylogenetic relationships among class 4 HDACs and the anomalous phyletic distribution of the eukaryotic ones: (i) ancient gene duplication followed by differential gene loss or (ii) horizontal gene transfer (HGT).

**Figure 3:**
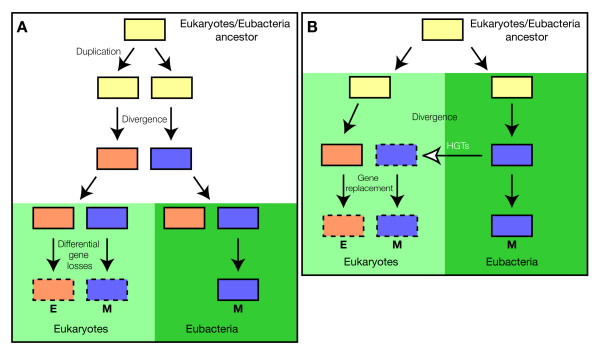
**Evolutionary history of class 4 HDACs**. Two possible evolutionary scenarios are represented. E, eukaryotic-group HDAC; M, mixed-group HDAC. Dotted lines indicate that the gene is not present in all eukaryotic species (see text for details).

Let us first examine the anomalous phyletic distribution of some eukaryotic class 4 HDACs in the light of the 'gene duplication-gene loss' hypothesis (Figure 3A). As both the mixed-group type and the eukaryotic-group type occur (often separately, sometimes together) in a wide range of eukaryotes, the duplication event postulated to have given rise to these two types of genes must have occurred early in eukaryotic evolution. The presence of two types of class 4 HDACs would thus be the ancestral situation of most or all eukaryotes. The punctate distribution we see today would be due to loss, at a high rate, of one or the other type of class 4 HDAC. As the mixed-group class 4 HDACs of eukaryotes appear more closely related to eubacterial HDACs than to the eukaryotic-group HDACs, we must even consider the possibility that the duplication event occurred before the eukaryotes and eubacteria diverged (Figure 3A). The presence of two types of class 4 HDACs would be the ancestral situation for both eubacteria and eukaryotes. The transition to the present-day situation would have involved not only a high rate of differential gene loss in eukaryotes, but also the loss of one of the paralogues in eubacteria, probably at an early stage of eubacterial evolution.

One main problem with this view is that both types of class 4 HDAC genes must have coexisted in the ancestors of lineages (e.g. metazoans and viridiplantae) where some descendants have one type of HDAC and other descendants have the other type. We would expect many of these organisms to still possess both genes, but as a rule, this is not so (Figure 2). We found both gene types in only three eukaryotic species, as opposed to 37 species possessing only one gene. As more genomes are sequenced, more will probably be found to contain both genes, but the presence of a single gene in most genomes studied to date does not support the notion that the two categories of class 4 HDACs represent two paralogous groups that originated early in eukaryotic evolution. In addition, the model of ancient gene duplication followed by differential gene loss fails to fully explain some of our observations, such as the strongly supported separation of two diatom HDACs, those of *Thalassiosira pseudonana *and *Phaeodactylum tricornutum*, within the mixed group (Figure 1). Much more complicated scenarios are therefore required, making this model less plausible and not especially parsimonious.

The other main possibility is HGT, the transmission of genetic material from one species to another (Figure 3B). HGT is a widespread and important phenomenon in prokaryotes. It is one of the driving forces of genome evolution in both archaea and eubacteria [13-18]. Over the past few years, it has become increasingly clear that HGT has had an impact on eukaryote evolution also, at least in the case of unicellular and/or parasitic eukaryotes [19-24], yet the occurrence and the importance of HGT in organisms such as land plants and animals is less obvious and very controversial. Although claims have been made for HGT in multicellular organisms, only very few cases have been clearly demonstrated, and these mainly concern eukaryote-eukaryote and/or host-parasite gene transfer [15,25-31]. The main criteria used in the aforementioned publications to detect HGT are unexpected phyletic distribution, differential presence or absence in closely related species, and incongruent phylogenetic trees [15,16,23,32]. Our data meet all these criteria (see Figures 1 and 2), and are therefore very suggestive of the occurrence of HGTs having shaped the evolutionary history of the class 4 HDACs.

Although our data do not allow a firm determination of the direction of these putative HGTs (the identity of donors and recipients remains unknown), we favour the hypothesis that transfer occurred from prokaryotes to eukaryotes, and that the eukaryotic-group members are the 'original' eukaryotic HDACs and the mixed-group members are the 'transferred' HDACS. In support of this view, class 4 HDACs are found in many diverse eubacterial species representative of most major eubacterial lineages (Figure 1). To imagine that a class 4 HDAC was present in early eubacterial evolution (and subsequently transferred a few times to eukaryotes) is a more parsimonious mechanism than to postulate that the different prokaryotic class 4 HDACs were acquired from eukaryotes by numerous independent HGTs.

An important feature of the putative prokaryote-eukaryote HGTs is that most of them are probably ancient, as indicated by the species ranges covered by the monophyletic groups distinguished among the mixed-group eukaryotic HDACs (Figure 1). For example, the existence of the aforementioned monophyletic group comprising all nine metazoan sequences indicates that the transferred gene was already present in the last common ancestor of these animals, i.e. in that of most or all animals. This means that the recipient of the putative HGT was not a present-day complex metazoan but an ancient, probably much more simple (maybe unicellular) ancestor. This is important, as gene transfers from prokaryotes to eukaryotes with sequestered germ lines, such as most present-day animals, appear to be very rare [23]; almost all other putative HGTs we have detected concern unicellular eukaryotes. A possible exception concerns the second HDAC sequence found in the genome of the cnidarian *Nematostella vectensis*, which forms a monophyletic group with the sequences of *Cytophaga hutchinsonii *and *Psychrobacter cryhalolentis *(Figure 1, orange circle). Although we cannot rule out contamination of the genomic data from which these sequences were obtained, this might be indicative of a much more recent HGT involving a complex multicellular organism.

Besides these putative eubacterium-eukaryote transfers, there is also the possibility of at least one eukaryote-eukaryote HGT. This is suggested by the existence of a monophyletic group including very distant eukaryotic species (Figure 1, yellow circle): the diatoms *Thalassiosira pseudonana *and *Phaeodactylum tricornutum *(chromalveolata), the red alga *Cyanidioschyon merolae*, and the green alga *Ostreococcus tauri *(plantae). The green alga sequence is more closely related to those of the evolutionarily very distant diatoms than to those of any other viridiplantae. We suggest that this association may be the result of eukaryote-eukaryote HGTs between these phytoplanctonic species.

Lastly, we note that in most lineages only a single HDAC is found (Figures 1 and 2), yet two different proteins are found in the diatoms *Thalassiosira pseudonana *and *Phaeodactylum tricornutum*, the cnidarian *Nematostella vectensis*, and the green alga *Ostreococcus tauri*. In all these cases, both proteins belong to the mixed group and are not closely related, suggesting independent HGTs. The existence of eukaryotes with only mixed-group HDACs (and thus lacking a eukaryotic-group member) suggests that gene transfer was sometimes followed by functional replacement of the 'original' eukaryotic gene by the transferred one. The only eukaryotes to possess both a mixed-group and a eukaryotic-group protein are the green alga *Chlamydomonas reinhardtii *and two animals (*Strongylocentrotus purpuratus *and *Locusta migratoria*) (Figures 1 and 2). Similar multiple replacements have been reported for the eukaryotic translation elongation factor 1α [21]. Whether these replacements are due solely to chance or have selective advantages [33] is an open question that awaits functional and biochemical characterization of the proteins and still broader sampling of eukaryotic HDAC genes.

## Conclusion

The results presented here shed new light on the evolutionary history of class 4 HDACs. These proteins display unexpected phylogenetic relationships, at odds with the phylogeny of the corresponding species, suggestive of ancient horizontal gene transfers between prokaryotes and eukaryotes. This suggests that the evolution of important eukaryotic multigene families, such as the histone deacetylase gene family, may have been shaped by horizontal gene transfers.

## Methods

Class 4 HDAC sequences were retrieved through BLAST searches [34] on protein and genome data, mainly from the NCBI [35], the Doe JGI [36], the Sanger Institute [37], the Baylor College of Medicine [38], the Genoscope [39], and the TIGR [40] databases. To ascertain that we identified class 4 HDACs, we first used the 'reciprocal best BLAST hit' criterion. We retained for each species only the best BLAST hits, using known class 4 HDACs (of animals) as queries. We then performed the reciprocal BLAST using the obtained sequences as queries against the NCBI NR database and verified that the class 4 HDACs initially used in the first BLAST search are the best BLAST hits in the corresponding species. All class 4 HDACs identified are listed in Additional File 2. As most of the identified sequences come from EST data and unfinished genomes, we were concerned about the possibility that some of them might represent contamination of the genomic data. We list our argument against this possibility in Additional File 6. In order to detect potential bacterial contaminations, we also performed an analysis of the codon usage of the HDAC coding sequences compared to the corresponding genomes. This analysis, which is shown in Additional Files 6 and 7, does not show any evidence for contamination.

Multiple alignments were performed with Clustal W [41] and subsequently manually improved. We performed two types of alignments, class 4 HDACs with HDACs of other classes and class 4 HDACs alone. The first type of alignment was used to verify the monophyly of the class 4 HDACs and thus to ascertain that we had identified *bona fide *class 4 HDACs (see Additional File 1). The second type of alignment was used to determine phylogenetic relationships among class 4 HDACs. In establishing the phylogeny of class 4 HDACs, we avoided using the first type of alignment (with other HDCAs serving as outgoups) to prevent potential phylogenetic reconstruction artefacts due to the presence of distant outliers (class 4 HDACs diverge considerably from other HDACs, not shown) [42]. We used both a multiple alignment containing the whole protein sequences and a multiple alignment containing only the regions with unequivocal alignment. Both alignments gave the same tree topologies. The alignment of the whole proteins (used to produce the trees shown in this paper) can be found in Additional File 8.

Unweighted maximum-parsimony (MP) and neighbour-joining (NJ) reconstructions were performed with the PAUP 4.0 program [43]. MP analyses were performed with the following settings: heuristic search of over 500 bootstrap replicates, MAXTREES set at 2000, and other parameters set at default values. Maximum likelihood (ML) analyses were performed with PHYML [44] and TreePuzzle [45]. PHYML analyses were performed using two different amino-acid substitution models, the Jones-Taylor-Thornton (JTT) model [46] and the Whelan and Goldman (WAG) model [47], the frequencies of amino acids being estimated from the data set, and rate heterogeneity across sites being modelled by two rate categories (one constant and eight γ rates). Statistical support for the different internal branches was assessed by bootstrap resampling (100 bootstrap replicates), as implemented in PHYML [44]. Bootstrap consensus trees were constructed with the PAUP 4.0 program. Treepuzzle analyses were performed by means of the quartet puzzling tree search procedure, with 25,000 puzzling steps [45]. We used the WAG model of substitution [47] and the frequencies of amino acids being estimated from the data set, and allowed rate heterogeneity across sites to be modelled by two rate categories (one constant and eight γ rates) [45]. Bayesian inference was performed using the Markov chain Monte Carlo method as implemented in the MRBAYES (version 3) package [48,49]. We used the WAG substitution frequency matrix [47] with among-sites rate variation modelled by means of a discrete γ distribution with four equally probable categories. Two independent Markov chains were run, each containing 1,000,000 Monte Carlo steps, after a burn-in of 400,000 steps. One out of every 100 trees was saved. For each run, we computed the majority consensus of the obtained trees by means of the PAUP 4.0 program. The same consensus tree was obtained for both runs. Marginal probabilities at each node were taken as a measure of statistical support. The discrepancy between the estimated probabilities obtained in the two runs was 5% on the average and never exceeded 11%. The results obtained from the two runs are thus consistent, so that we finally combined them by gathering the trees of both samples.

## Authors' contributions

VL and MV conceived the study, retrieved the sequences used in the study, and made the sequence alignments. MV performed the phylogenetic analysis. VL carried out the analysis of codon usage. Both authors participated in the writing and editing of the manuscript. Both authors read and approved the final manuscript.

## Supplementary Material

Additional File 1 **Monophyly of the class 4 HDACs**. **(A) **Phylogenetic analysis of the class 4 HDACs with the other (human) HDACs. The class 4 HDACs form a well-supported monophyletic group. The tree is an MP bootstrap consensus tree. **(B) **Phylogenetic analysis of the class 4 HDACs with a sample of class 1 HDACs (which are the HDACs closest to the class 4 HDACs). The class 4 HDACs form a well-supported monophyletic group. The tree is an NJ bootstrap consensus tree. **(C) **Phylogenetic analysis of the class 4 HDACs with a large set of class-1 and class-2 HDACs, including the divergent archaeal HDACs. The class 4 HDACs form a well-supported monophyletic group. The tree is an NJ bootstrap consensus tree. For the three trees, the different statistical support values are as in Figure 1, rooting is arbitrary, and the colour code of the class 4 HDACS is as in Figure 1. Click here for file

Additional File 2 **Table 1: List of all the class 4 HDACs used in the phylogenetic analyses**. Asterisks denote partial sequences that come from unassembled genomes (from the NCBI Trace Archive) or EST data. All other sequences are full-length proteins that have been deduced from fully-sequenced assembled genomes and/or cloned cDNAs. Click here for file

Additional File 3 **Phylogenetic tree of the class 4 HDACs as determined by maximum likelihood analysis**. The tree was generated with PHYML using the WAG amino-acid substitution model. Numbers above the branches are bootstrap support values obtained using the WAG and JTT models, respectively. The colour code of species names is as in Figure 1. Rooting is arbitrary. Click here for file

Additional File 4 **Phylogenetic tree of the class 4 HDACs as determined by Bayesian inference**. The tree (majority rule consensus tree) was generated with MRBAYES. Numbers above the branches are posterior probabilities. The colour code of species names is as in Figure 1. Rooting is arbitrary. Click here for file

Additional File 5 **Phylogenetic tree of the class 4 HDACs as determined by maximum parsimony analysis**. The tree (bootstrap consensus tree) was generated with PAUP. Numbers above the branches are bootstrap support values. The colour code of species names is as in Figure 1. Rooting is arbitrary. Click here for file

Additional File 6 Arguments against the possibility that the eukaryotic class 4 HDACs belonging to the "mixed group" are bacterial contamination. Click here for file

Additional File 7 **Codon usage of 68 class 4 HDAC coding sequences with respect to the corresponding genomes**. Pearson's χ^2 ^test was performed to measure the goodness of fit between the observed codon usage distribution of 68 class 4 HDAC coding sequences and those expected for 69 different genomes. Several applicability conditions were tested, in which a minimum of 0, 1, 2, and 5 codon occurrences was mandatory. The highest number of consistence between a given class 4 HDAC and its source genome is observed for thresholds set to 0 or 1. During each test, stop codons were not taken into account, and codons that did not fulfil the applicability conditions were discarded from the analysis. A *p*-value was calculated for each Class 4 HDAC/genome tested. A *p*-value close to zero signals that the null hypothesis is false, and typically that a difference is very likely to exist between compared class 4 HDAC and genome codon usages. Large *p*-values closer to 1 imply that there is no detectable difference for the sample size used. A "NA" label replaces the "source genome *p*-value" and *p*-value ratio of class 4 HDACs for which there were no corresponding genome codon usage table available and too few known coding sequences to derive a significative codon usage table. The source genome table we took for the Picea engelmannii HDAC is the available codon usage table of the closely-related species, *Picea abies*. Click here for file

Additional File 8 Multiple alignment of the class 4 HDACs. Click here for file
