# Supplementary material for: Comparative genomics of the class 4 histone deacetylase family indicates a complex evolutionary history
Source: BMC Biol. 2006 Aug 2;4:24. doi: 10.1186/1741-7007-4-24 (PMC1555614; doi:10.1186/1741-7007-4-24)
Supplement: Additional File 1 — Monophyly of the class 4 HDACs. (A) Phylogenetic analysis of the class 4 HDACs with the other (human) HDACs. The class 4 HDACs form a well-supported monophyletic group. The tree is an MP bootstrap consensus tree. (B) Phylogenetic analysis of the class 4 HDACs with a sample of class 1 HDACs (which are the HDACs closest to the class 4 HDACs). The class 4 HDACs form a well-supported monophyletic group. The tree is an NJ bootstrap consensus tree. (C) Phylogenetic analysis of the class 4 HDACs with a large set of class-1 and class-2 HDACs, including the divergent archaeal HDACs. The class 4 HDACs form a well-supported monophyletic group. The tree is an NJ bootstrap consensus tree. For the three trees, the different statistical support values are as in Figure 1, rooting is arbitrary, and the colour code of the class 4 HDACS is as in Figure 1. [file 1741-7007-4-24-S1.pdf]

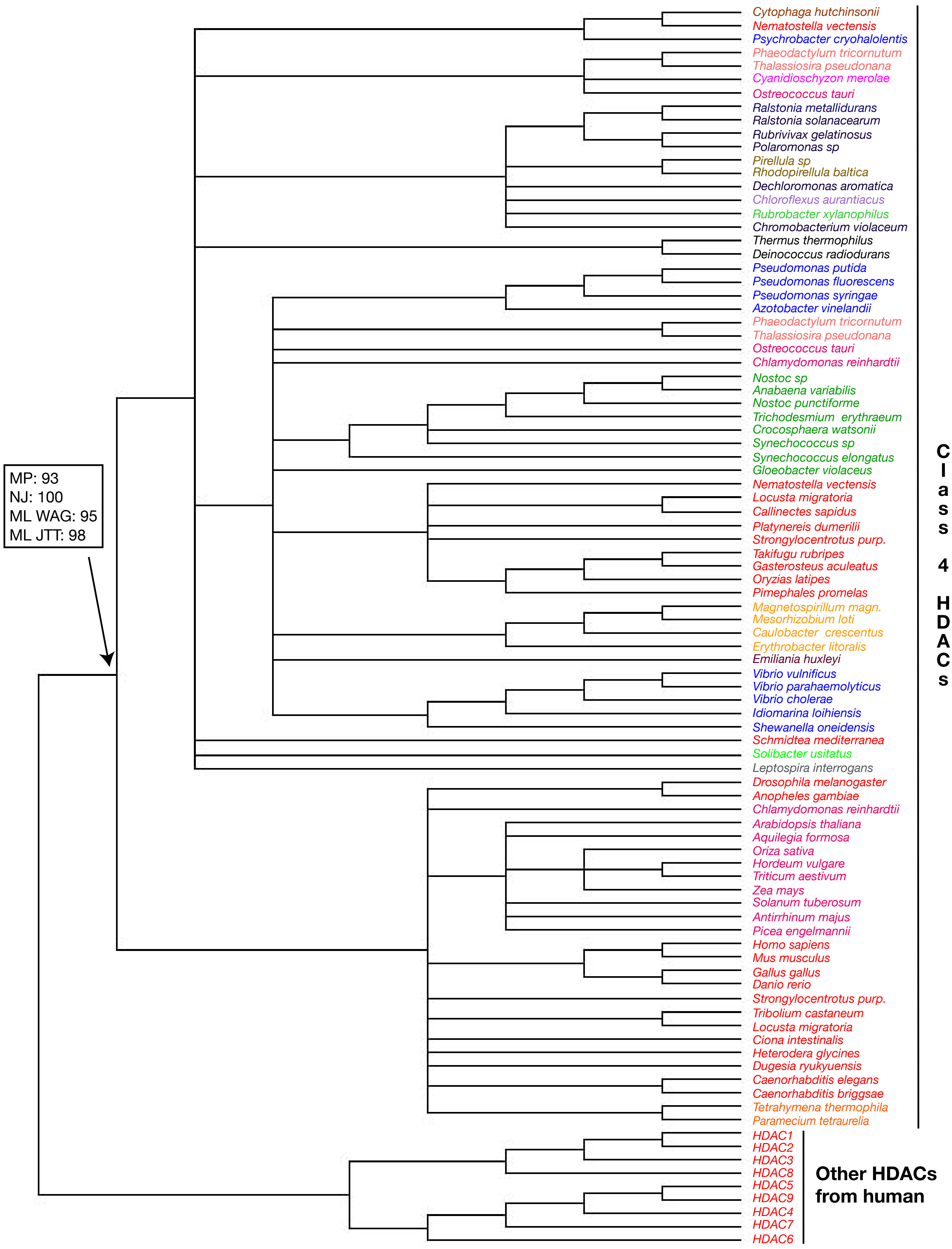
Cytophaga hutchinsonii
Nematostella vectensis
Psychrobacter cryohalolentis
Phaeodactylum tricornutum
Thalassiosira pseudonana
Cyanidioschyzon merolae
Ostreococcus tauri
Ralstonia metallidurans
Ralstonia solanacearum
Rubrivivax gelatinosus
Polaromonas sp
Pirellula sp
Rhodopirellula baltica
Dechloromonas aromatica
Chloroflexus aurantiacus
Rubrobacter xylanophilus
Chromobacterium violaceum
Thermus thermophilus
Deinococcus radiodurans
Pseudomonas putida
Pseudomonas fluorescens
Pseudomonas syringae
Azotobacter vinelandii
Phaeodactylum tricornutum
Thalassiosira pseudonana
Ostreococcus tauri
Chlamydomonas reinhardtii
Nostoc sp
Anabaena variabilis
Nostoc punctiforme
Trichodesmium erythraeum
Crocosphaera watsonii
Synechococcus sp
Synechococcus elongatus
Gloeobacter violaceus
Nematostella vectensis
Locusta migratoria
Callinectes sapidus
Platynereis dumerilii
Strongylocentrotus purp.
Takifugu rubripes
Gasterosteus aculeatus
Oryzias latipes
Pimephales promelas
Magnetospirillum magn.
Mesorhizobium loti
Caulobacter crescentus
Erythrobacter litoralis
Emiliania huxleyi
Vibrio vulnificus
Vibrio parahaemolyticus
Vibrio cholerae
Idiomarina loihiensis
Shewanella oneidensis
Schmidtea mediterranea
Solibacter usitatus
Leptospira interrogans
Drosophila melanogaster
Anopheles gambiae
Chlamydomonas reinhardtii
Arabidopsis thaliana
Aquilegia formosa
Oriza sativa
Hordeum vulgare
Triticum aestivum
Zea mays
Solanum tuberosum
Antirrhinum majus
Picea engelmannii
Homo sapiens
Mus musculus
Gallus gallus
Danio rerio
Strongylocentrotus purp.
Tribolium castaneum
Locusta migratoria
Ciona intestinalis
Heterodera glycines
Dugesia ryukyuensis
Caenorhabditis elegans
Caenorhabditis briggsae
Tetrahymena thermophila
Paramecium tetraurelia
HDAC1
HDAC2
HDAC3
HDAC8
HDAC5
HDAC9
HDAC4
HDAC7
HDAC6
MP: 93
NJ: 100
ML WAG: 95
ML JTT: 98
Class 4 HDACs
Other HDACs from human

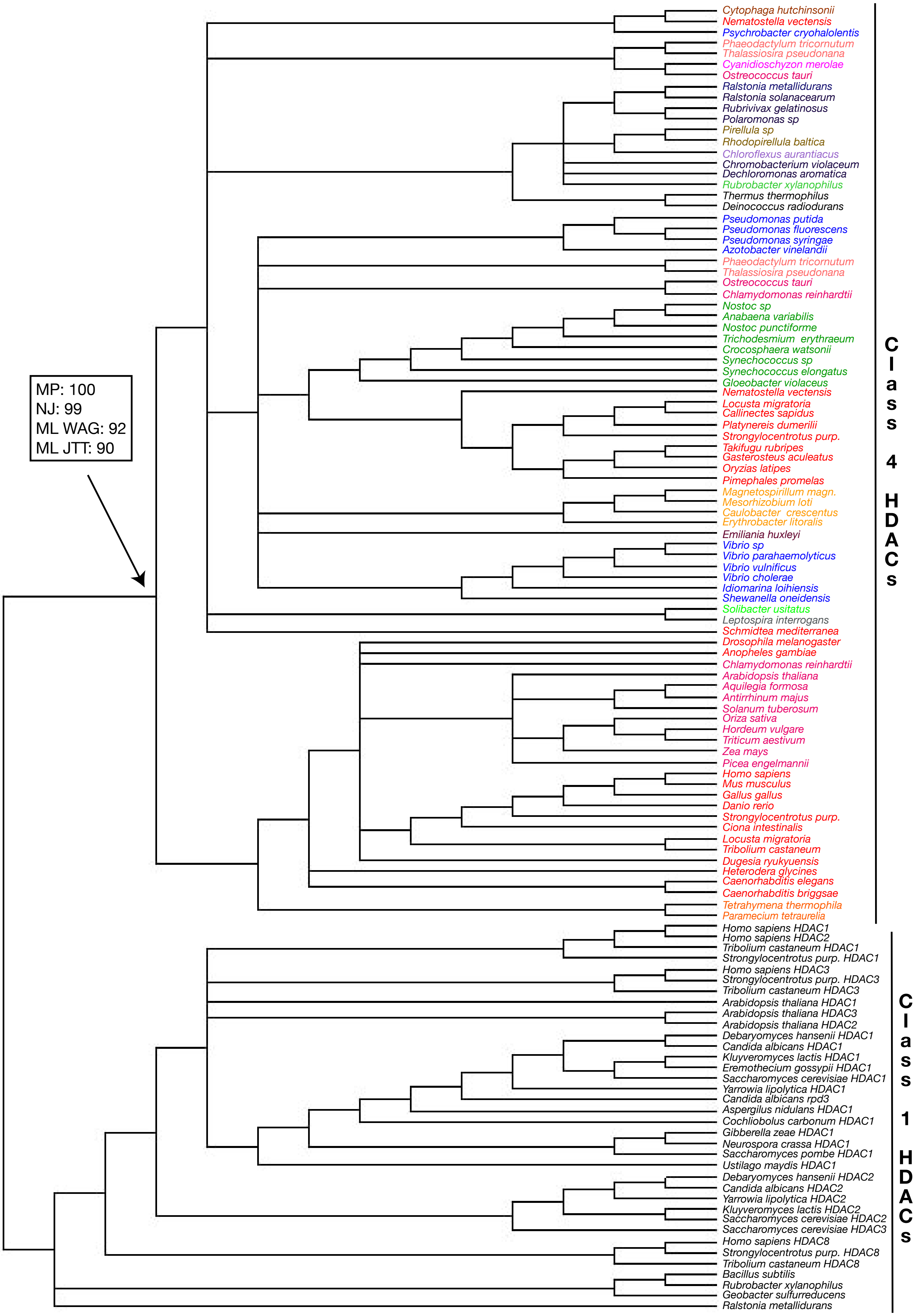

MP: 100
NJ: 99
ML WAG: 92
ML JTT: 90
Cytophaga hutchinsonii
Nematostella vectensis
Psychrobacter cryohalolentis
Phaeodactylum tricornutum
Thalassiosira pseudonana
Cyanidioschyzon merolae
Ostreococcus tauri
Ralstonia metallidurans
Ralstonia solanacearum
Rubrivivax gelatinosus
Polaromonas sp
Pirellula sp
Rhodopirellula baltica
Chloroflexus aurantiacus
Chromobacterium violaceum
Dechloromonas aromatica
Rubrobacter xylanophilus
Thermus thermophilus
Deinococcus radiodurans
Pseudomonas putida
Pseudomonas fluorescens
Pseudomonas syringae
Azotobacter vinelandii
Phaeodactylum tricornutum
Thalassiosira pseudonana
Ostreococcus tauri
Chlamydomonas reinhardtii
Nostoc sp
Anabaena variabilis
Nostoc punctiforme
Trichodesmium erythraeum
Crocosphaera watsonii
Synechococcus sp
Synechococcus elongatus
Gloeobacter violaceus
Nematostella vectensis
Locusta migratoria
Callinectes sapidus
Platynereis dumerilii
Strongylocentrotus purp.
Takifugu rubripes
Gasterosteus aculeatus
Oryzias latipes
Pimephales promelas
Magnetospirillum magn.
Mesorhizobium loti
Caulobacter crescentus
Erythrobacter litoralis
Emiliania huxleyi
Vibrio sp
Vibrio parahaemolyticus
Vibrio vulnificus
Vibrio cholerae
Idiomarina loihiensis
Shewanella oneidensis
Solibacter usitatus
Leptospira interrogans
Schmidtea mediterranea
Drosophila melanogaster
Anopheles gambiae
Chlamydomonas reinhardtii
Arabidopsis thaliana
Aquilegia formosa
Antirrhinum majus
Solanum tuberosum
Oriza sativa
Hordeum vulgare
Triticum aestivum
Zea mays
Picea engelmannii
Homo sapiens
Mus musculus
Gallus gallus
Danio rerio
Strongylocentrotus purp.
Ciona intestinalis
Locusta migratoria
Tribolium castaneum
Dugesia ryukyuensis
Heterodera glycines
Caenorhabditis elegans
Caenorhabditis briggsae
Tetrahymena thermophila
Paramecium tetraurelia
Class 4 HDACs
Homo sapiens HDAC1
Homo sapiens HDAC2
Tribolium castaneum HDAC1
Strongylocentrotus purp. HDAC1
Homo sapiens HDAC3
Strongylocentrotus purp. HDAC3
Tribolium castaneum HDAC3
Arabidopsis thaliana HDAC1
Arabidopsis thaliana HDAC3
Arabidopsis thaliana HDAC2
Debaryomyces hansenii HDAC1
Candida albicans HDAC1
Kluyveromyces lactis HDAC1
Eremothecium gossypii HDAC1
Saccharomyces cerevisiae HDAC1
Yarrowia lipolytica HDAC1
Candida albicans rpd3
Aspergilus nidulans HDAC1
Cochliobolus carbonum HDAC1
Gibberella zeae HDAC1
Neurospora crassa HDAC1
Saccharomyces pombe HDAC1
Ustilago maydis HDAC1
Debaryomyces hansenii HDAC2
Candida albicans HDAC2
Yarrowia lipolytica HDAC2
Kluyveromyces lactis HDAC2
Saccharomyces cerevisiae HDAC2
Saccharomyces cerevisiae HDAC3
Homo sapiens HDAC8
Strongylocentrotus purp. HDAC8
Tribolium castaneum HDAC8
Bacillus subtilis
Rubrobacter xylanophilus
Geobacter sulfurreducens
Ralstonia metallidurans
Class 1 HDACs

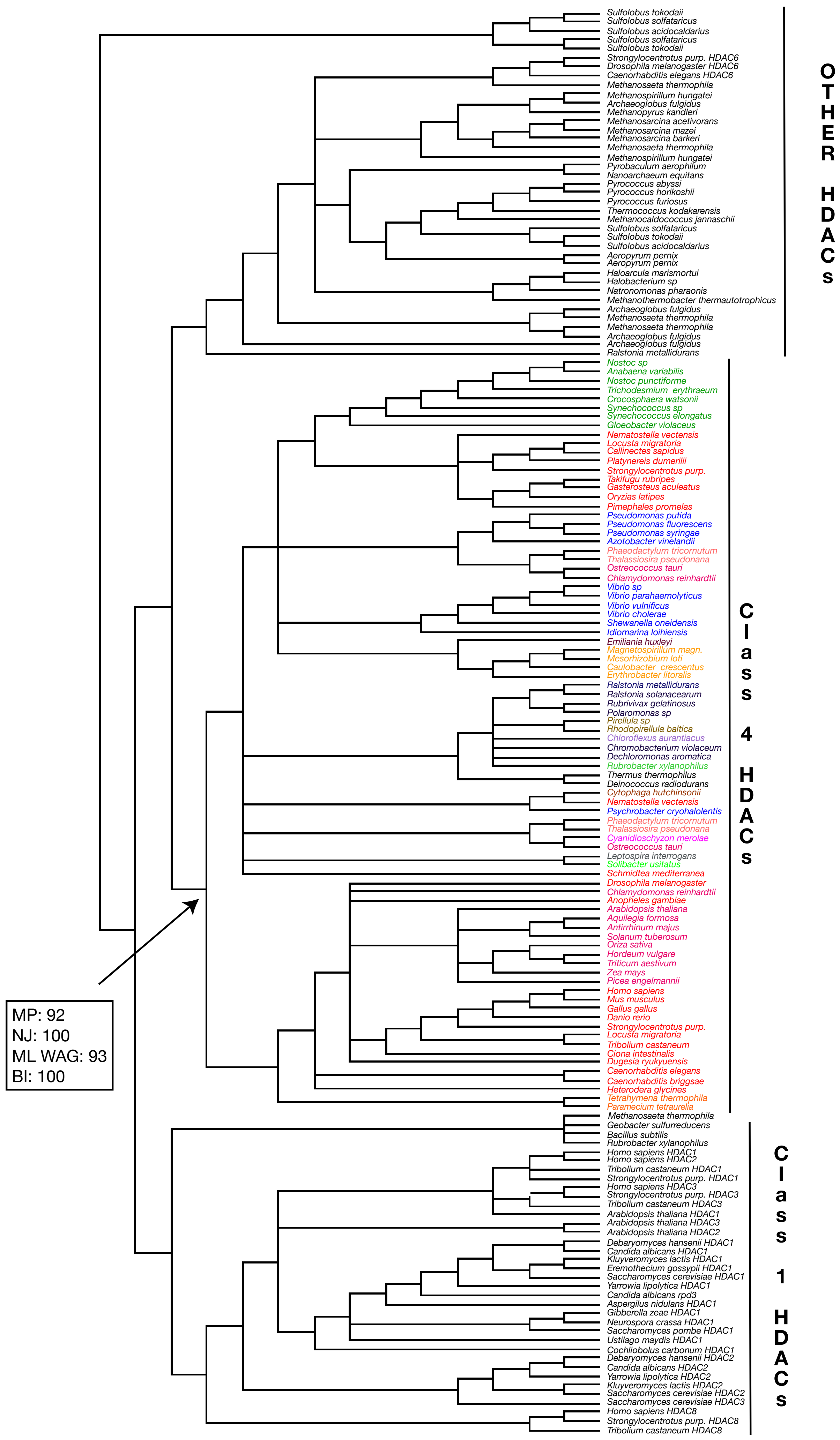
Sulfolobus tokodaii
Sulfolobus solfataricus
Sulfolobus acidocaldarius
Sulfolobus solfataricus
Sulfolobus tokodaii
Strongylocentrotus purp. HDAC6
Drosophila melanogaster HDAC6
Caenorhabditis elegans HDAC6
Methanosaeta thermophila
Methanospirillum hungatei
Archaeoglobus fulgidus
Methanopyrus kandleri
Methanosarcina acetivorans
Methanosarcina mazei
Methanosarcina barkeri
Methanosaeta thermophila
Methanospirillum hungatei
Pyrobaculum aerophilum
Nanoarchaeum equitans
Pyrococcus abyssi
Pyrococcus horikoshii
Pyrococcus furiosus
Thermococcus kodakarensis
Methanocaldococcus jannaschii
Sulfolobus solfataricus
Sulfolobus tokodaii
Sulfolobus acidocaldarius
Aeropyrum pernix
Aeropyrum pernix
Haloarcula marismortui
Halobacterium sp
Natronomonas pharaonis
Methanothermobacter thermautotrophicus
Archaeoglobus fulgidus
Methanosaeta thermophila
Methanosaeta thermophila
Archaeoglobus fulgidus
Archaeoglobus fulgidus
Ralstonia metallidurans
OTHER HDACs
Nostoc sp
Anabaena variabilis
Nostoc punctiforme
Trichodesmium erythraeum
Crocosphaera watsonii
Synechococcus sp
Synechococcus elongatus
Gloeobacter violaceus
Nematostella vectensis
Locusta migratoria
Callinectes sapidus
Platynereis dumerilii
Strongylocentrotus purp.
Takifugu rubripes
Gasterosteus aculeatus
Oryzias latipes
Pimephales promelas
Pseudomonas putida
Pseudomonas fluorescens
Pseudomonas syringae
Azotobacter vinelandii
Phaeodactylum tricornutum
Thalassiosira pseudonana
Ostreococcus tauri
Chlamydomonas reinhardtii
Vibrio sp
Vibrio parahaemolyticus
Vibrio vulnificus
Vibrio cholerae
Shewanella oneidensis
Idiomarina loihiensis
Emiliania huxleyi
Magnetospirillum magn.
Mesorhizobium loti
Caulobacter crescentus
Erythrobacter litoralis
Ralstonia metallidurans
Ralstonia solanacearum
Rubrivivax gelatinosus
Polaromonas sp
Pirellula sp
Rhodopirellula baltica
Chloroflexus aurantiacus
Chromobacterium violaceum
Dechloromonas aromatica
Rubrobacter xylanophilus
Thermus thermophilus
Deinococcus radiodurans
Cytophaga hutchinsonii
Nematostella vectensis
Psychrobacter cryohalolentis
Phaeodactylum tricornutum
Thalassiosira pseudonana
Cyanidioschyzon merolae
Ostreococcus tauri
Leptospira interrogans
Solibacter usitatus
Schmidtea mediterranea
Drosophila melanogaster
Chlamydomonas reinhardtii
Anopheles gambiae
Arabidopsis thaliana
Aquilegia formosa
Antirrhinum majus
Solanum tuberosum
Oriza sativa
Hordeum vulgare
Triticum aestivum
Zea mays
Picea engelmannii
Homo sapiens
Mus musculus
Gallus gallus
Danio rerio
Strongylocentrotus purp.
Locusta migratoria
Tribolium castaneum
Ciona intestinalis
Dugesia ryukyuensis
Caenorhabditis elegans
Caenorhabditis briggsae
Heterodera glycines
Tetrahymena thermophila
Paramecium tetraurelia
Class 4 HDACs
MP: 92
NJ: 100
ML WAG: 93
BI: 100
Methanosaeta thermophila
Geobacter sulfurreducens
Bacillus subtilis
Rubrobacter xylanophilus
Homo sapiens HDAC1
Homo sapiens HDAC2
Tribolium castaneum HDAC1
Strongylocentrotus purp. HDAC1
Homo sapiens HDAC3
Strongylocentrotus purp. HDAC3
Tribolium castaneum HDAC3
Arabidopsis thaliana HDAC1
Arabidopsis thaliana HDAC3
Arabidopsis thaliana HDAC2
Debaryomyces hansenii HDAC1
Candida albicans HDAC1
Kluyveromyces lactis HDAC1
Eremothecium gossypii HDAC1
Saccharomyces cerevisiae HDAC1
Yarrowia lipolytica HDAC1
Candida albicans rpd3
Aspergillus nidulans HDAC1
Gibberella zeae HDAC1
Neurospora crassa HDAC1
Saccharomyces pombe HDAC1
Ustilago maydis HDAC1
Cochliobolus carbonum HDAC1
Debaryomyces hansenii HDAC2
Candida albicans HDAC2
Yarrowia lipolytica HDAC2
Kluyveromyces lactis HDAC2
Saccharomyces cerevisiae HDAC2
Saccharomyces cerevisiae HDAC3
Homo sapiens HDAC8
Strongylocentrotus purp. HDAC8
Tribolium castaneum HDAC8
Class 1 HDACs
