# Supplementary material for: Comparative genomics of the class 4 histone deacetylase family indicates a complex evolutionary history
Source: BMC Biol. 2006 Aug 2;4:24. doi: 10.1186/1741-7007-4-24 (PMC1555614; doi:10.1186/1741-7007-4-24)
Supplement: Additional File 2 — Table 1: List of all the class 4 HDACs used in the phylogenetic analyses. Asterisks denote partial sequences that come from unassembled genomes (from the NCBI Trace Archive) or EST data. All other sequences are full-length proteins that have been deduced from fully-sequenced assembled genomes and/or cloned cDNAs. [file 1741-7007-4-24-S2.pdf]

Table 1: List of all the class 4 HDACs used in the phylogenetic analyses.

(a) Prokaryotic class 4 HDACs.

**Cyanobacteria**	Nostocales	*Nostoc sp*	ref|NP_487390.1|
		Anabaena variabilis	gb|ABA22800.1|
		Nostoc punctiforme	ref|ZP_00108329.1|
	Oscillatoriales	*Trichodesmium erythraeum*	ref|ZP_00326307.1|
	Chroococcales	*Crocosphaera watsonii*	ref|ZP_00175036.1|
		Synechococcus sp	emb|CAA78368.1|
		Synechococcus elongatus	ref|ZP_00164477.1|
	Gloeobacteria	*Gloeobacter violaceus*	ref|NP_924565.1|
**α-proteobacteria**	Rhodospirillales	*Magnetospirillum magn.*	ref|ZP_00048995.1|
	Rhizobiales	*Mesorhizobium loti*	ref|NP_106295.1|
	Caulobacterales	*Caulobacter crescentus*	ref|NP_422442.1|
	Sphingomonadales	*Erythrobacter litoralis*	ref|ZP_00375703.1|
**γ-proteobacteria**	Pseudomonadales	*Pseudomonas putida*	ref|NP_746870.1|
		Pseudomonas fluorescens	ref|ZP_00262636.1|
		Pseudomonas syringae	ref|ZP_00205602.1|
		Azotobacter vinelandii	ref|ZP_00089457.1|
		Psychrobacter cryohalolentis	ref|ZP_00203918.1|
	Vibrionales	*Vibrio vulnificus*	ref|NP_761923.1|
		Vibrio parahaemolyticus	ref|NP_798524.1|
		Vibrio cholerae	ref|NP_231676.1|
	Alteromonadales	*Shewanella oneidensis*	ref|NP_717423.1|
		Idiomarina loihiensis	ref|YP_156242.1|
**β-proteobacteria**	Burkholderiales	*Ralstonia metallidurans*	ref|ZP_00274142.1|
		Ralstonia solanacearum	ref|NP_519727.1|
		Rubrivivax gelatinosus	ref|ZP_00243804.1|
		Polaromonas sp	ref|ZP_00507823.1|
	Rhodocyclales	*Dechloromonas aromatica*	ref|ZP_00151420.1|
	Neisseriales	*Chromobacterium violaceum*	ref|NP_901996.1|
**Acidobacteria**	Solibacteres	*Solibacter usitatus*	ref|ZP_00525925.1|
**Planctomycetes**	Planctomycetacia	*Pirellula sp*	ref|NP_870264.1|
		Rhodopirellula baltica	emb|CAD77339.1|
**Chloroflexi**	Chloroflexales	*Chloroflexus aurantiacus*	ref|ZP_00020099.1|
**Actinobacteria**	Rubrobacteridae	*Rubrobacter xylanophilus*	ref|ZP_00199957.1|
**Deinococcus-Thermus**	Deinococci	*Thermus thermophilus*	ref|YP_005923.1|
		Deinococcus radiodurans	ref|NP_294557.1|
**Bacteroidetes**	Sphingobacteria	*Cytophaga hutchinsonii*	ref|ZP_00309925.1|
**Spirochaetes**	Spirochaetales	*Leptospira interrogans*	ref|NP_713103.1|

(b) Eukaryotic class 4 HDACs.

**Metazoa**	Cnidaria	*Nematostella vectensis*	gblATWA420234.1| *<br>gb|DV092267.1| *
	Arthropoda	*Tribolium castaneum*	gb|CM000279.1|
		Anopheles gambiae	ref|XP_321350.1|
		Drosophila melanogaster	ref|NP_733048.1|
		Locusta migratoria	gb|CO826273.1| *<br>gb|CO837592.1| *
		Callinectes sapidus	gb|CV480200.1| *
	Nematoda	*Caenorhabditis briggsae*	emb|CAE64834.1|
		Caenorhabditis elegans	emb|CAA94910.2|
		Heterodera glycines	gb|CA939397.1| *
	Platyhelminthes	*Schmidtea mediterranea*	gb| AY967490.1| *
		Dugesia ryukyuensis	dbj|BW641523.1| *
	Annelida	*Platynereis dumerilii*	emb|AM265393.1|
	Echinodermata	*Strongylocentrotus purpuratus*	ref|XP_784008.1| ref|XP_783282.1|
	Chordata	*Homo sapiens*	sp|Q96DB2|
		Mus musculus	ref|NP_659168.1|
		Gallus gallus	ref|XP_414321.1|
		Danio rerio	ref|NP_00100217.1|
		Takifugu rubripes	emb|CAAB01000023.1|
		Gasterosteus aculeatus	gb|DN729245.1| *
		Pimephales promelas	gb|DT315240.1| *
		Oryzias latipes	dbj|BJ019457.1| *
		Ciona intestinalis	dbj|AK173892.1|
**Viridiplantae**	Streptophyta	*Arabidopsis thaliana*	ref|NP_568480.2|
		Solanum tuberosum	gb|BG595396.1| *
		Antirrhinum majus	emb|AJ795636.1| *
		Aquilegia formosa	gb|DR950703.1| *
		Triticum aestivum	gb|CV771496.1| *
		Zea mays	gb|DR809014.1| *
		Oriza sativa	dbj|BAD61930.1|
		Hordeum vulgare	gb|BU991783.1| *
		Picea engelmannii	gb|DR469846.1| *
	Chlorophyta	*Ostreococcus tauri*	lcl|Ot16g01260 chrom16.0001 *<br>lcl|Ot16g02010 chrom16.0001
		Chlamydomonas reinhardtii	C_110047 [chlre2:153873]<br>C_850021 [chlre2:170585]
**Rhodophyta**	Bangiophyceae	*Cyanidioschyzon merolae*	gnl|CMER|CMQ158C|
**Stramenopiles**	Bacillariophyta	*Phaeodactylum tricornutum*	gblAWSB540267.1| *<br>gblATSB76748.1| *
		Thalassiosira pseudonana	newV2.0.genewise.125.2.1 [thaps1:164714]<br>newV2.0.genewise.116.35.1 [thaps1:117338]
**Haptophyceae**	Isochrysidales	*Emiliania huxleyi*	gb|AKBS156999.g2| *
**Alveolata**	Ciliophora	*Tetrahymena thermophila*	gb|CH445712.1| *
		Paramecium tetraurelia	GWSUNIT00006285001
