# Supplementary figures and images for: Comparative genomics of the class 4 histone deacetylase family indicates a complex evolutionary history
Source: BMC Biol. 2006 Aug 2;4:24. doi: 10.1186/1741-7007-4-24 (PMC1555614; doi:10.1186/1741-7007-4-24)

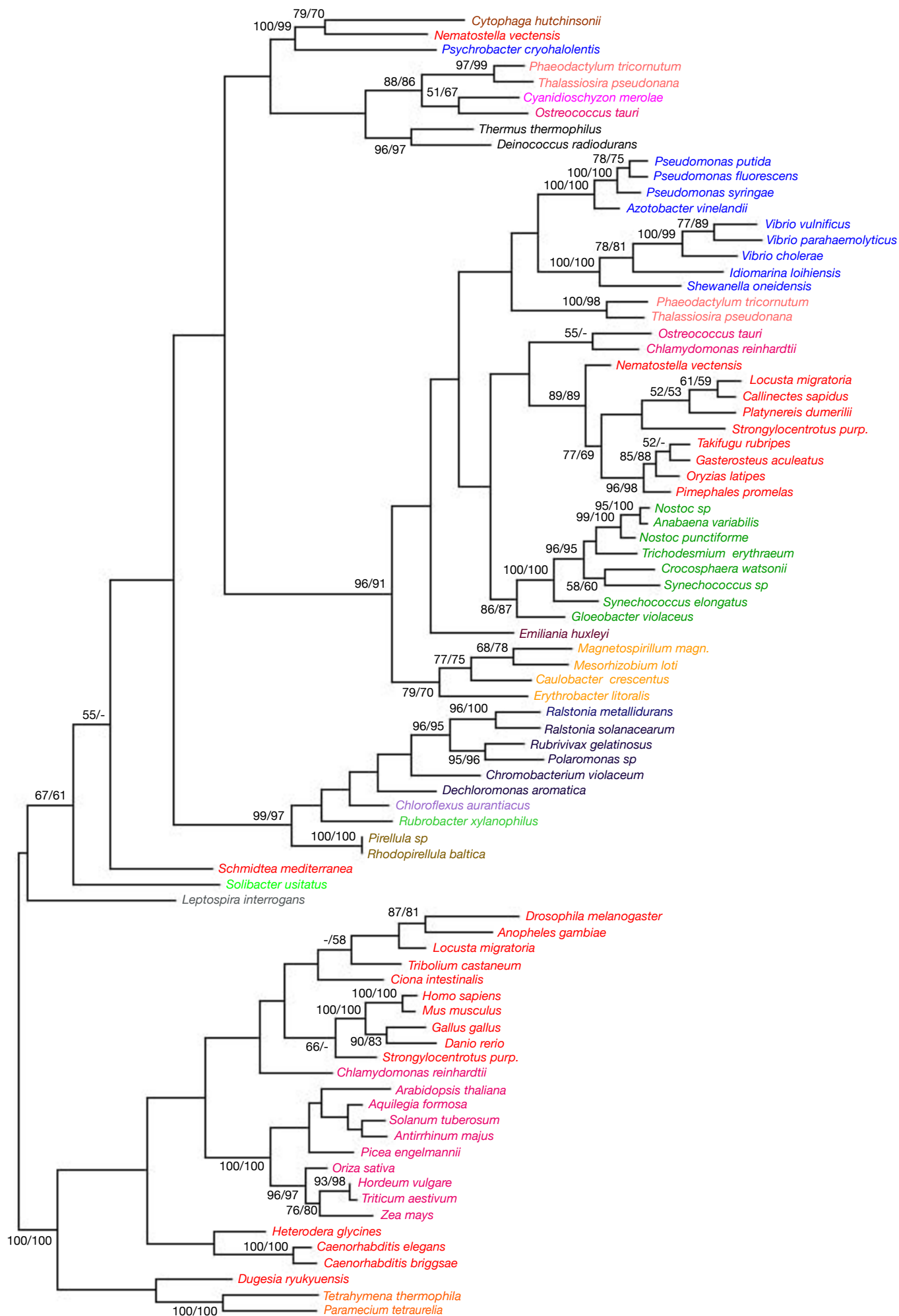

Supplement: Additional File 3 — Phylogenetic tree of the class 4 HDACs as determined by maximum likelihood analysis. The tree was generated with PHYML using the WAG amino-acid substitution model. Numbers above the branches are bootstrap support values obtained using the WAG and JTT models, respectively. The colour code of species names is as in Figure 1. Rooting is arbitrary. [file 1741-7007-4-24-S3.pdf]

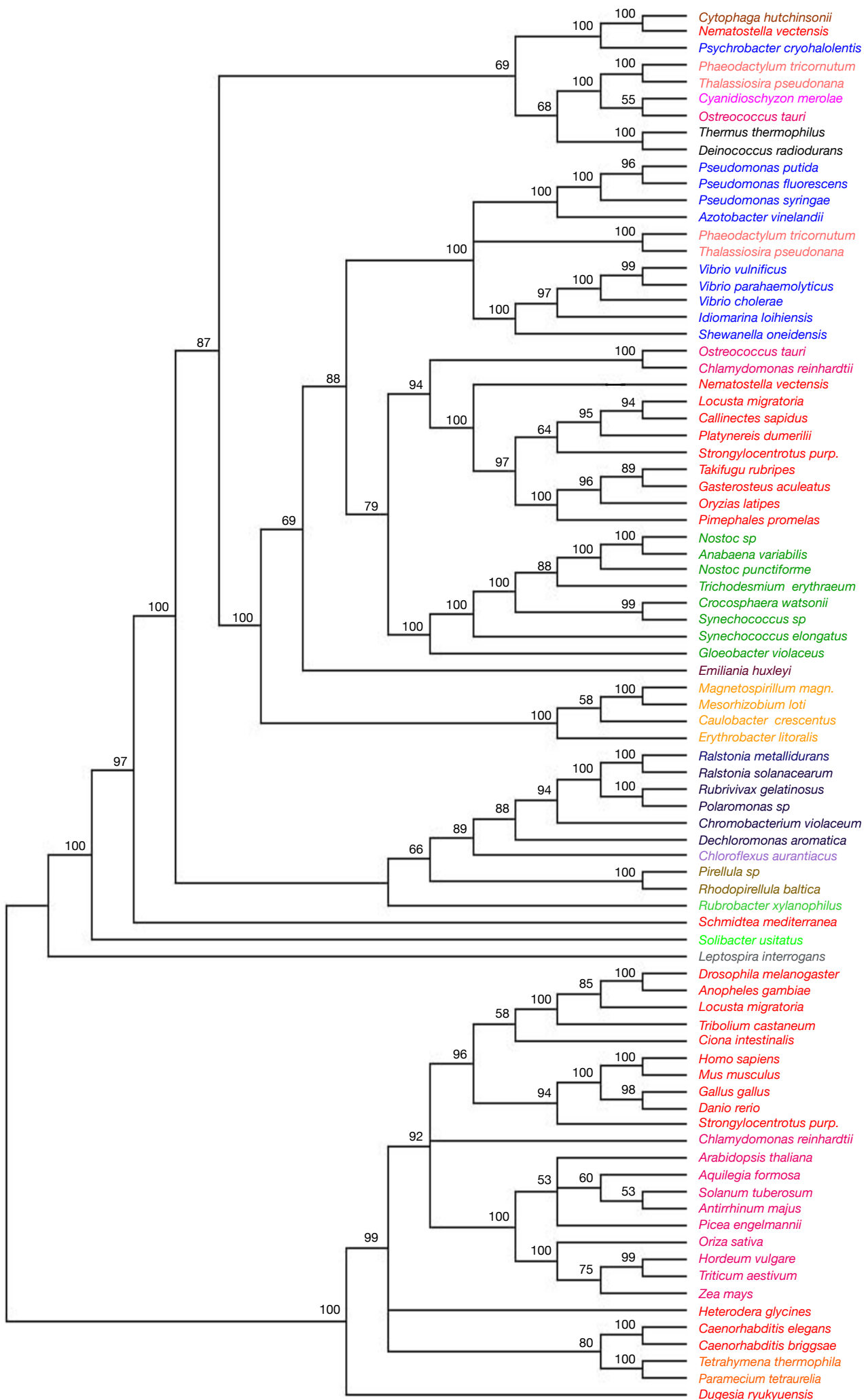

Supplement: Additional File 4 — Phylogenetic tree of the class 4 HDACs as determined by Bayesian inference. The tree (majority rule consensus tree) was generated with MRBAYES. Numbers above the branches are posterior probabilities. The colour code of species names is as in Figure 1. Rooting is arbitrary. [file 1741-7007-4-24-S4.pdf]

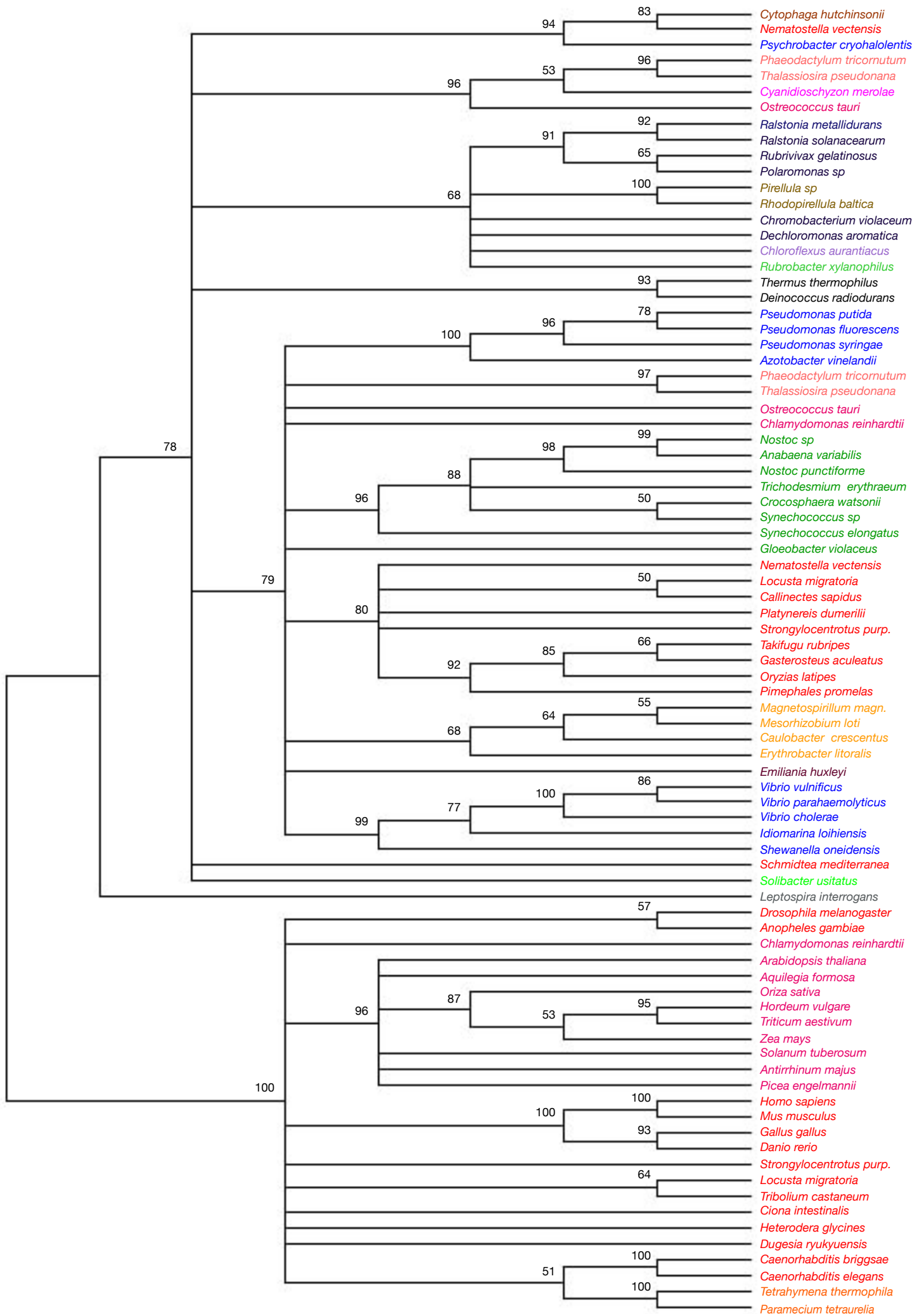

Supplement: Additional File 5 — Phylogenetic tree of the class 4 HDACs as determined by maximum parsimony analysis. The tree (bootstrap consensus tree) was generated with PAUP. Numbers above the branches are bootstrap support values. The colour code of species names is as in Figure 1. Rooting is arbitrary. [file 1741-7007-4-24-S5.pdf]
