# Supplementary material for: Comparative genomics of the class 4 histone deacetylase family indicates a complex evolutionary history
Source: BMC Biol. 2006 Aug 2;4:24. doi: 10.1186/1741-7007-4-24 (PMC1555614; doi:10.1186/1741-7007-4-24)
Supplement: Additional File 6 — Arguments against the possibility that the eukaryotic class 4 HDACs belonging to the "mixed group" are bacterial contamination. [file 1741-7007-4-24-S6.pdf]

We want to discuss here the possibility that the eukaryotic HDACs that belong to the mixed group (whose sequence mainly come from EST data or Whole Genome Shotgun (WGS) reads) may result from contaminations of the libraries used for the sequencing and may therefore be eubacterial proteins. We used four different approaches to assert the effective presence of the HDACs in the genomes of several species used in our analyses, that all rule out the hypothesis of contamination.

First, among the eukaryotic genomes in which we have found a class 4 HDAC from the mixed group, there are *Fugu rubripes*, *Strongylocentrotus purpuratus*, the diatom *Thalassiosira pseudonana*, as well as *Chlamydomonas reinhardtii* for which genome assembling has been done. In the species, the HDAC genes are not preliminary sequences and are included in genomic contigs containing eukaryotic genes. Moreover, in the case of *Fugu rubripes* and *Strongylocentrotus purpuratus*, the HDACs are encoded by spliced genes, as determined in the genome annotation of these two species.

Second, in the case of *Platynereis dumerilii*, we have confirmed the existence of the HDAC sequence by RT-PCR on mRNA from different larval stages (not shown).

Third, and most importantly, most of the eukaryotic HDACs that are in the mixed group do form monophyletic groups inside this clade. If we consider for instance the nine animal sequences that group together inside the mixed group, the possibility of a contamination would imply that, just by chance, the different sequences (some are WGS reads, other ESTs) obtained in different sequencing centres from animals with much different life styles and habitats would have been contaminated by different bacteria that are much closer to each other than to any other known bacteria. It would also imply that, by chance, the bacteria that would have contaminated the DNA libraries of the four fishes are more closely-related to each other than to those that would have contaminated the other libraries. This does not seem to us a parsimonious and proper interpretation of the observation of this monophyletic group. This holds true also for most other eukaryotic HDACs, as they also form monophyletic groups with other eukaryotic sequences.

Finally, we tried to determine whether the codon usage of the eukaryotic HDACs from the mixed group is consistent with the codon usage of eukaryotic organisms and inconsistent with the closest apparent bacterial relatives. A summary of the obtained results is shown in Additional file 7. Tables representative of genomes codon usage were retrieved at the Codon Usage Database [1] (for file names with extension .cutg), the NRCUD [2] (for file names with extension .cud), or were created by retrieving the coding regions of available genomes at the NCBI (for file names with extension .cusp), and treating them with the program cusp for creation of codon usage tables, EMBOSS package version 3.0.0 [3]. The nucleic sequences

coding for Class 4 HDAC were retrieved mainly at the NCBI, the Doe JGI, the Sanger Institute, the Baylor College of Medecine, and the TIGR. Codon usage tables of a representative set of 69 class 4 coding sequences were created thanks to the program cusp of the EMBOSS package version 3.0.0 [3]. We performed Pearson's Chi-squared tests implemented in the R package version 2.3.0 [4] to measure the agreement between the observed codon usage distribution of 68 class 4 HDAC coding sequences and those expected for 69 different genomes. Stop codons were not taken into account and several applicability conditions were tested in which a minimum of 0, 1, 2 and 5 codon occurrences was mandatory. During each test, codons that did not fulfil the conditions were discarded from the analysis. A p-value was calculated for each Class 4 HDAC/genome tested, the Null Hypothesis statement being that no difference is observed between the codon usage distributions of the class 4 HDAC and genome tested. A p-value close to zero signals that the null hypothesis is false, and typically that a difference is very likely to exist. Large p-values closer to 1 imply that there is no detectable difference for the sample size used. We found that, in many cases, the best match of the codon usage of a given HDAC coding sequence is not the codon usage of the corresponding genome. This is true for both eukaryotic (from both the mixed group and eukaryotic group) and eubacterial HDACs. Nevertheless, in most cases, the eubacterial HDAC coding sequences match better with eubacterial genomes and eukaryotic HDAC coding sequences match better with eukaryotic genomes. This is true for both the HDACs from the mixed and eukaryotic groups. For example the codon usage of the HDACs coding sequences from *Platynereis dumerilii*, *Fugu rubripes*, *Locusta migratoria*, *Callinectes sapidus*, and *Strongylocentrotus purpuratus*, which belong to the « mixed group », have a best match with the codon usage of eukaryotic genomes. This represents an additional argument against the possibility that these sequences represent bacterial contaminations. There are a few cases in which an eukaryotic HDAC coding sequence has a best match with an eubacterial genome. For example, in the analysis made with a treshold set to a minimum of 1 codon occurrence, the codon usage of the HDAC coding sequence from *Dugesia ryukyuensis* (which belongs to the eukaryotic group) has a best match with the codon usage of the genome of *Trichodesmium erythraeum* ; the codon usage of the HDAC coding sequence from *Gasterosteus aculeatus* (which belongs to the mixed group) has a best match with the codon usage of the genome of *Rubrobacter xylanophilus*. We do not know the signification of this, as the HDACs from *Dugesia* and *Trichodesmium* are not associated in the phylogenetic analysis, nor are those from *Gasterosteus* and *Rubrobacter*. In addition, in some cases, the best match is dependent of the treshold used for the analysis : when the treshold is set to 0, the HDAC coding sequence from *Gasterosteus aculeatus* has a best match

with the codon usage of the genome of *Homo sapiens*. Our conclusion is that analysis of codon usages does not provide any evidence for bacterial contaminations and further indicate that most of the eukaryotic HDACs from the mixed group really are eukaryotic sequences.

We therefore conclude that the anomalous phyletic distribution of some eukaryotic class 4 HDACs is not an artefact due to eubacterial contaminations of the DNA materials used for sequencing.

References

1. **The Codon Usage Database** [http://www.kazusa.or.jp/codon/]

2. **The Non-redundant Codon Usage Database.** [http://bioinformatics.forsyth.org/nrcud/index.php /]

3. Rice, P., Longden, I. and Bleasby,A.: **EMBOSS: The European Molecular Biology Open Software Suite.** *Trends in Genetics* 2000, **16** (6): pp276—277

4. The R project for Statistical Computing [http://www.r-project.org/index.html]
