# Supplementary material for: Comparative genomics of the class 4 histone deacetylase family indicates a complex evolutionary history
Source: BMC Biol. 2006 Aug 2;4:24. doi: 10.1186/1741-7007-4-24 (PMC1555614; doi:10.1186/1741-7007-4-24)
Supplement: Additional File 8 — Multiple alignment of the class 4 HDACs. [file 1741-7007-4-24-S8.pdf]

```
Alignment: /Users/michelvervoort/Desktop/hdacf3.paup
Seaview [blocks=10 fontsize=9 A4] on Fri May 12 17:05:51 2006

                              1
Cytophaga hutchinsonii        ---------- ---------- ---------- ---------- ---------- ----------
Nematostella vectensis        ---------- ---------- ---------- ---------- ---------- ----------
Psychrobacter cryohalolentis  ---------- ---------- ---------- ---------- ---------- ----------
Phaeodactylum tricornutum     ---------- ---------- ---------- ---------- ---------- ----------
Thalassiosira pseudonana      ---------- ---------- ---------- ---------- ---------- ----------
Cyanidioschyzon merolae       ---------- ---------- ---------- ---------- ---------- ----------
Ostreococcus tauri            ---------- ---------- ---------- ---------- ---------- ----------
Ralstonia metallidurans       ---------- ---------- ---------- ---------- ---------- ----------
Ralstonia solanacearum        ---------- ---------- ---------- ---------- ---------- ----------
Rubrivivax gelatinosus        ---------- ---------- ---------- ---------- ---------- ----------
Polaromonas sp                ---------- ---------- ---------- ---------- ---------- ----------
Pirellula sp                  ---------- ---------- ---------- ---------- ---------- ----------
Rhodopirellula baltica        ---------- ---------- ---------- ---------- ---------- ----------
Chloroflexus aurantiacus      ---------- ---------- ---------- ---------- ---------- ----------
Dechloromonas aromatica       ---------- ---------- ---------- ---------- ---------- ----------
Chromobacterium violaceum     ---------- ---------- ---------- ---------- ---------- ----------
Rubrobacter xylanophilus      ---------- ---------- ---------- ---------- ---------- ----------
Thermus thermophilus          ---------- ---------- ---------- ---------- ---------- ----------
Deinococcus radiodurans       ---------- ---------- ---------- ---------- ---------- ----------
Pseudomonas putida            ---------- ---------- ---------- ---------- ---------- ----------
Pseudomonas fluorescens       ---------- ---------- ---------- ---------- ---------- ----------
Pseudomonas syringae          ---------- ---------- ---------- ---------- ---------- ----------
Azotobacter vinelandii        ---------- ---------- ---------- ---------- ---------- ----------
Phaeodactylum tricornutum     ---------- ---------- ---------- ---------- ---------- ----------
Thalassiosira pseudonana      ---------- ---------- ---------- ---------- ---------- ----------
Ostreococcus tauri            ---------- ---------- ---------- ---------- ---------- ----------
Chlamydomonas reinhardtii     ---------- ---------- ---------- ---------- ---------- ----------
Nostoc sp                     ---------- ---------- ---------- ---------- ---------- ----------
Anabaena variabilis           ---------- ---------- ---------- ---------- ---------- ----------
Nostoc punctiforme            ---------- ---------- ---------- ---------- ---------- ----------
Trichodesmium  erythraeum     ---------- ---------- ---------- ---------- ---------- ----------
Crocosphaera watsonii         ---------- ---------- ---------- ---------- ---------- ----------
Synechococcus sp              ---------- ---------- ---------- ---------- ---------- ----------
Synechococcus elongatus       ---------- ---------- ---------- ---------- ---------- ----------
Gloeobacter violaceus         ---------- ---------- ---------- ---------- ---------- ----------
Nematostella vectensis        ---------- ---------- ---------- ---------- ---------- ----------
Locusta migratoria            ---------- ---------- ---------- ---------- ---------- ----------
Callinectes sapidus           ---------- ---------- ---------- ---------- ---------- ----------
Platynereis dumerilii         ---------- ---------- ---------- ---------- ---------- ----------
Strongylocentrotus purp.      ---------- ---------- ---------- ---------- -----MLAYG WSWGPRHGAQ
Takifugu rubripes             ---------- ---------- ---------- ---------- ---------- ----------
Oryzias latipes               ---------- ---------- ---------- ---------- ---------- ----------
Gasterosteus aculeatus        ---------- ---------- ---------- ---------- ---------- ----------
Pimephales promelas           ---------- ---------- ---------- ---------- ---------- ----------
Magnetospirillum magn.        ---------- ---------- ---------- ---------- ---------- ----------
Mesorhizobium loti            ---------- ---------- ---------- ---------- ---------- ----------
Caulobacter crescentus        ---------- ---------- ---------- ---------- ---------- ----------
Erythrobacter litoralis       ---------- ---------- ---------- ---------- ---------- ----------
Emiliania huxleyi             ---------- ---------- ---------- ---------- ---------- ----------
Vibrio vulnificus             ---------- ---------- ---------- ---------- ---------- ----------
Vibrio parahaemolyticus       ---------- ---------- ---------- ---------- ---------- ----------
Vibrio cholerae               ---------- ---------- ---------- ---------- ---------- ----------
Shewanella oneidensis         ---------- ---------- ---------- ---------- ---------- ----------
Idiomarina loihiensis         ---------- ---------- ---------- ---------- ---------- ----------
Solibacter usitatus           ---------- ---------- ---------- ---------- ---------- ----------
Schmidtea mediterranea        ---------- ---------- ---------- ---------- ---------- ----------
Drosophila melanogaster       ---------- ---------- ---------- ---------- ---------- ---------M
Chlamydomonas reinhardtii     ---------- ---------- ---------- ---------- ---------- ----------
Anopheles gambiae             ---------- ---------- ---KGISRWP RKCVSADDLP WFAQSSGMCF RLNSTASADQ
Arabidopsis thaliana          ---------- --------MT HTRVISTWTE LTRDLAIYLL FTFFAIKVFK FLFSCNRTSE
Aquilegia formosa             ---------- ---------- ---------- ---------- ---------- ----------
Oriza sativa                  ---------- ---------- ---------- ---------- ---------- -----MASSA
Hordeum vulgare               ---------- ---------- ---------- ---------- ---------- ----------
Picea engelmannii             ---------- ---------- ---------- ---------- ---------- ----------
Homo sapiens                  ---------- ---------- ---------- ---------- ---------- ----------
Mus musculus                  ---------- ---------- ---------- ---------- ---------- ----------
Gallus gallus                 ---------- ---------- ---------- ---------- ---------- -GAGGAGPRG
Danio rerio                   ---------- ---------- ---------- ---------- ---------- ----------
Strongylocentrotus purp.      ---------- ---------- ---------- ---------- ---------- ----------
Tribolium castaneum           ---------- ---------- ---------- ---------- ---------- ----------
Locusta migratoria            ---------- ---------- ---------- ---------- ---------- ----------
Ciona intestinalis            ---------- ---------- ---------- ---------- ---------- ----------
Dugesia ryukyuensis           ---------- ---------- ---------- ---------- ---------- ----------
Solanum tuberosum             ---------- ---------- ---------- ---------- ---------- -----SPDVV
Antirrhinum majus             ---------- ---------- ---------- ---------- -----LSLTA KA---TSSTK
Triticum aestivum             AYLGDTHRTS LYKKAGWYRS GIPGISSTHA SGDRETIRAP -----LSIPA RALGMSSSSA
Zea mays                      --------RR ILSRHRNRCS SARNARAPQP ISHRRIPRPP RPFGFSPIPA RRHGTSPSLS
Heterodera glycines           ---------- ---------- ---------- ---------- --------LI KAFHLLISLP
Caenorhabditis briggsae       ---------- ---------- ---------- ---------- ---------- ----------
Caenorhabditis elegans        ---------- ---------- ---------- ---------- ---------- ----------
Tetrahymena thermophila       ---------- ---------M YTLICAVQDL ALFGIFITGL VLLEWQIIVF PILIYILIPF
Paramecium tetraurelia        ---------- -------MQL YTIIDWFFTI GSLVLAILLG VYVNGAACLL GVFLYFIYPF
Leptospira interrogans        ---------- ---------- ---------- ---------- ---------- ----------
```

```
                                61
Cytophaga hutchinsonii          ---------- ---------- ---------- --------ML QIAYHPVYNH PV---PEGHR
Nematostella vectensis          ---------- ---------- ---------- ---------- ---------- ----------
Psychrobacter cryohalolentis    ---------- ---------- ---------- --------ML KIAYSDIFRY SV---PEKHR
Phaeodactylum tricornutum       ---------- ---------- ---------- ---------- -IYYNDVYEV KL---PKNHR
Thalassiosira pseudonana        ---------- ---------- ---------- ---------- ---------- ----------
Cyanidioschyzon merolae         ---------- ---------- ---------- ---------- -------YAV EL---PAGHR
Ostreococcus tauri              ---------- ---------- ---------- ---------- -VYHADWARV DL---PDGHR
Ralstonia metallidurans         ---------- ---------- ---------- ---------M LAFYADHFVL PL---PPGHR
Ralstonia solanacearum          ---------- ---------- ---------- -------MLM QAFYTDHFVL PL---PPGHR
Rubrivivax gelatinosus          ---------- ---------- ---------- ---------M HAYYSDHYVL PL---PAGHR
Polaromonas sp                  ---------- ------MATR RCALAVATGI FEHLPGQPML QAFYADNFVL PL---PPGHR
Pirellula sp                    ---------- ---------- ---------- ---------M RLYYTDHFDL PL---PEGHR
Rhodopirellula baltica          ---------- ---------- ---------- ---------M RLYYTDHFDL PL---PEGHR
Chloroflexus aurantiacus        ---------- ---------- ---------- ---------M RAFYSDTFVL PL---PPGHR
Dechloromonas aromatica         ---------- ---------- ---------- ---------M RLFYTDVFVL PL---PAGHR
Chromobacterium violaceum       ---------- ---------- ---------- ---------M RIYRTDQFPL PL---PAGHR
Rubrobacter xylanophilus        ---------- ---------- ---------- ---------M RVFYSDSYVI PL---PEGHR
Thermus thermophilus            ---------- ---------- ---------- ---------M RAYTTAHLSL PL---PEGHP
Deinococcus radiodurans         ---------- ---------- ---------- -----MAHPF RAYSPADFHF PL---PEGHR
Pseudomonas putida              ---------- ---------- ---------- -------MPL PLIYHEDYSP E---FPAEHR
Pseudomonas fluorescens         ---------- ---------- ---------- -------MPL PLIYHEDYSP E---FPADHR
Pseudomonas syringae            ---------- ---------- ---------- -------MSL PLIYHEDYSP D---FPADHR
Azotobacter vinelandii          ---------- ---------- -------MNR PLPRSAAMSL PLVYHDEYSP S---FPDGHR
Phaeodactylum tricornutum       ---------- ---------- ---------- ---------- -IVYHEDYSF AD--WPSLHT
Thalassiosira pseudonana        ---------- ---------- ---------- ---------- ---------- ----------
Ostreococcus tauri              ---------- ---------- ---------- ARAPGAGGRV PVVHHDSYSA PT--LPRGHR
Chlamydomonas reinhardtii       ---------- ---------- ---------- ---------- PVVYHPDYSA P---------
Nostoc sp                       ---------- ---------- ---------- -------MEL PIIYHPDYVA PL---PEGHR
Anabaena variabilis             ---------- ---------- ---------- -------MEL PIIYHPHYVA PL---PEGHR
Nostoc punctiforme              ---------- ---------- ---------- -------MDL PIIYHPDYIA PL---PEGHR
Trichodesmium  erythraeum       ---------- ---------- ---------- -------MNL PVIYHPNYVA PI---PPGHR
Crocosphaera watsonii           ---------- ---------- ---------- -------MNL PIVYDHQYVV PL---PNGHR
Synechococcus sp                ---------- ---------- ---------- -------MGF PVVYHPDYVT PI---PEEHR
Synechococcus elongatus         ---------- ---------- ---------- -------MDL PLVYHPGYSA PL---PSTHR
Gloeobacter violaceus           ---------- ---------- ---------- -------MEL PFVYSPRYEA NL---PPAHR
Nematostella vectensis          ---------- ---------- ---------- ---------- ---------- ----------
Locusta migratoria              ---------- ---------- ---------- ---------- ---------- ----------
Callinectes sapidus             ---------- ---------- ---------- ---------- ---------- ----------
Platynereis dumerilii           ---------- ---------- MRSQFIRTFA ARTTVTSNCL PIIHHHDYVS EL---PKGHR
Strongylocentrotus purp.        ARLGGQARIH GGQNYTPASV VFHTSKPDKE TPHISNAPNL PIVHHDGYES YL---PPGHR
Takifugu rubripes               ---------- ---------- ---------- ---------L PIIHHSKYVC DL---PTNHR
Oryzias latipes                 ---------- ---------V RNGLCRCFHA EHVRLESSGL PIIHHSKYVC EL---PANHR
Gasterosteus aculeatus          ---------- ---------- -------FHT EPVRHESSAL PIVHHSKYVC DL---PPNHR
Pimephales promelas             ---------- ---------- ---------- ---------- ---------- ----------
Magnetospirillum magn.          ---------- ---------- ---------- ---------- --MFHPAYEA AL---PAGHR
Mesorhizobium loti              ---------- ---------- ---------- -------MPL QIVHHPDYDA GF---ATNHR
Caulobacter crescentus          ---------- ---------- ---------- -----MSAPP PIVHHPAFRA EM---PAGHR
Erythrobacter litoralis         ---------- ---------- ---------- ---------- --------MA PR---PERGT
Emiliania huxleyi               ---------- ---------- -------HKS QLHTASSAEV AWVYSREYAV LDG-WDSGHR
Vibrio vulnificus               ---------- ---------- ---------- ------MMSI PLIYHPIYSQ LP--LPEGHR
Vibrio parahaemolyticus         ---------- ---------- ---------- --------ML PLIYHSIYSR LE--LPEGHR
Vibrio cholerae                 ---------- ---------- ---------- --------MI PLIYHPIYSQ LD--LPVGHR
Shewanella oneidensis           ---------- ---------- ---------- --------MI PLVYHASYSK LA--LPIHHR
Idiomarina loihiensis           ---------- ---------- ---------- ----MKKVDV PFIYSPLYSE LD--LPPRHR
Solibacter usitatus             ---------- ---------- ---------- ------MLPY RFVYHDRYDL NLG-E---HV
Schmidtea mediterranea          ---------- ---------- --------TF INYHXTMSNY KLFYSPYYYA EIG-EG--HV
Drosophila melanogaster         -----KRLTE R---VHHEEQ ES-EEVVWVK RDDARSAGKL PIVFSRNYAV RFGGLERLHP
Chlamydomonas reinhardtii       ---------- ---------- ---------- -----PRLHL PPPPPPQYNI SFFGIEKLHP
Anopheles gambiae               -----SSMSS G---TDEDDK EDEAAGGKLT AELPIGRDRI PIVYRPEYGV RFLGLQKLHP
Arabidopsis thaliana            -----ISSFS M---ATHPEA LRRERILNSK LYFDVPLSKV SIIYSSSYDI SFMGIEKLHP
Aquilegia formosa               ---------- ---------- ---------- ---------- ---------- ----------
Oriza sativa                    -----PSAAA G---ATPPDP LRRDRILSSK LYLDVPGSKA PVVYSPAYDI AFLGIEKLHP
Hordeum vulgare                 ---------- ---------- ---------- ---------- ---------- ----------
Picea engelmannii               ---------- ---------- ---------- ---------- ---------- ----------
Homo sapiens                    ---------- ---------- ----MLHTTQ LYQHVPETRW PIVYSPRYNI TFMGLEKLHP
Mus musculus                    ---------- ---------- ----MPHATQ LYQHVPEKRW PIVYSPRYNI TFMGLEKLHP
Gallus gallus                   -----DPSPA R---PRGAPR PGAGMPHRTE LYEGVPPTCW PIVYSPDYNI TFMGLEKLHP
Danio rerio                     ---------- M---SKKEDS GGKKCSHHTE LYAEVPASCL PIVYSPEYNI TFMGLEKLHP
Strongylocentrotus purp.        ---------- ---------- --------SK LYVNVEPHQW PIIYSPDYNI SFLGFEKLHP
Tribolium castaneum             ---------- ---------- -------STK LYVDIGPEQW PIIYRPEYNV RFLGLEKLHP
Locusta migratoria              ---------- ---------- ---------- ---------- ---------- ----------
Ciona intestinalis              ---------- ---------- ---------- ---------- ---------- ----------
Dugesia ryukyuensis             ---------- ---------- ---------- ---------- ---------- ----------
Solanum tuberosum               -----PASSS K---TDDAEA LRRQRILSSH LYYDVPPSKV PLIYSPSYDI AFFGIEKLHP
Antirrhinum majus               -----SSNSS Q---NDDVDP VRLNRIRSSN LYFDVPTSKV PLIYSSCYDI AFLGIEKLHP
Triticum aestivum               -----AASVP G---ATPADA LRRNRIISSK LYFDVPGSKA PVVYSTAYDI AFLGIEKMHP
Zea mays                        RWRAMASSSS SPAPALAGEA LRQKRILSSK LYLEVPSSKA PVVYSPAYDI SFLGLEKLHP
Heterodera glycines             ------MSSS A---ALSAFA FLDD----SE AEIDWD-ERC PIVYSEDYNI SFFGIEKCHP
Caenorhabditis briggsae         ---------- ---MAEREE- ---------E ENYNLEEHQR PIVYHSEYNV SAFGIEHLHP
Caenorhabditis elegans          ---------- ---------- ---------- ---------- ---------- ----------
Tetrahymena thermophila         LNELVYNCFA KLLFTIEKEK KRMEQRYSQS ELENKYIENL PILYHPSYNI TACGIEKLHP
Paramecium tetraurelia          IMEFSYN--- --LLSVR--- ---------E EICIRNMPEI PIVYDESYNI TACGIEKWHP
Leptospira interrogans          ---------- ---------- ---------- --MEKHLERI GLVYHPDYNL DLG---P-HV
```

```
                                 121
Cytophaga hutchinsonii           FPMLKYDLIA EQLV------ --YEGTITE- ---------- ---------- ----------
Nematostella vectensis           ---------- ---------- ---EETIEA- ---------- ---------- ----------
Psychrobacter cryohalolentis     FPMQKYTMIP ERLL------ --AEKTISA- ---------- ---------- ----------
Phaeodactylum tricornutum        FPMEKYGRVR QLVQQWLQNL PVDEQGVVN- ---------- ---------- ----------
Thalassiosira pseudonana         ---------- ---------- ---------- ---------- ---------- ----------
Cyanidioschyzon merolae          FPMAKYELVR RKLE------ -ADSSFKG-- ---------- ---------- ----------
Ostreococcus tauri               FPMDKYQLAR LALQ------ -NDDTLRGK- ---------- ---------- ----------
Ralstonia metallidurans          FPMRKYSMLR DAVV------ -REVGG--V- ---------- ---------- ----------
Ralstonia solanacearum           FPMRKYSLLR ERAL------ -AEVPG--L- ---------- ---------- ----------
Rubrivivax gelatinosus           FPMAKYRMLR DRLA------ -AELPA--L- ---------- ---------- ----------
Polaromonas sp                   FPMGKYKLLR DRLI------ -SELPH--V- ---------- ---------- ----------
Pirellula sp                     FPMSKYRLLR QRVV------ -ESEHHRDD- ---------- ---------- ----------
Rhodopirellula baltica           FPMSKYRLLR QRVV------ -ESEHHRDD- ---------- ---------- ----------
Chloroflexus aurantiacus         FPMEKYALLR ERVL------ -SEGIVTPE- ---------- ---------- ----------
Dechloromonas aromatica          FPMEKYSRLR QALL------ -ASGEFSES- ---------- ---------- ----------
Chromobacterium violaceum        FPAEKYRLLA EQVS------ -AFAAER--- ---------- ---------- ----------
Rubrobacter xylanophilus         FPMRKYAMLR ERVA------ -AGRASG--- ---------- ---------- ----------
Thermus thermophilus             FPLYKYGGVA EALK------ -GLLPV---- ---------- ---------- ----------
Deinococcus radiodurans          FPYYKYEGVR ARLT------ -GLLPI---- ---------- ---------- ----------
Pseudomonas putida               FPMDKFRLLH DHLV------ -GSGLTT--- ---------- ---------- ---------D
Pseudomonas fluorescens          FPMDKFRLLR DHLV------ -DSGLTR--- ---------- ---------- ---------D
Pseudomonas syringae             FPMDKFRLLR DYLI------ -DSGLTS--- ---------- ---------- ---------D
Azotobacter vinelandii           FPMEKFRLLR DHLV------ -DSGLTS--- ---------- ---------- ---------D
Phaeodactylum tricornutum        FPMDKFARTA HALL------ -TTCKATYP- ---------- ---------- ---------E
Thalassiosira pseudonana         ---------- ---------- ---------- ---------- ---------- ----------
Ostreococcus tauri               FPMGVFQRVR DALA------ -REGIVRVG- ---------- ---------- ---------D
Chlamydomonas reinhardtii        ---------- ---------- ---------- ---------- ---------- ----------
Nostoc sp                        FPMAKFKKLY ELLL------ -SDDVAQTE- ---------- ---------- ----------
Anabaena variabilis              FPMAKFKKLY ELLL------ -SDDVAQTE- ---------- ---------- ----------
Nostoc punctiforme               FPMSKFRQLY ELLL------ -ADGVANQE- ---------- ---------- ----------
Trichodesmium  erythraeum        FPMAKFQLLY EMLL------ -VDEVTN--- ---------- ---------- ----------
Crocosphaera watsonii            FPMGKFSLLY ELLL------ -KEKIIDFN- ---------- ---------- ----------
Synechococcus sp                 FPMPKFRLLH GLLL------ -EDGVIQPE- ---------- ---------- ----------
Synechococcus elongatus          FPMAKFRLLH DRLL------ -RERVVYPQ- ---------- ---------- ----------
Gloeobacter violaceus            FPMGKFSRLH HYLL------ -NQGVARPE- ---------- ---------- ----------
Nematostella vectensis           ---------- ---------- ---------- ---------- ---------- ----------
Locusta migratoria               ---------- --LL------ -SDGIISLK- ---------- ---------- ----------
Callinectes sapidus              ---------- ---L------ -RDGVIKKN- ---------- ---------- ----------
Platynereis dumerilii            FAMKKFHGVF NYLL------ -KDNIIQM-- ---------- ---------- ----------
Strongylocentrotus purp.         FQMRKFNKLR DVLL------ -HDGVISKKQ MSSPVHVTKD ELIRVHTEEY IEKFFEGKTS
Takifugu rubripes                FPMGKFPRVL HFLF------ -KDQVITENQ ---------- ---------- ----------
Oryzias latipes                  FPMGKFPRVL HFLL------ -KDQVITEKQ V--------- ---------- ----------
Gasterosteus aculeatus           FPMAKFPRVL HHLI------ -KDQVITEKQ V--------- ---------- ----------
Pimephales promelas              ---------- ---------- ---------- ---------- ---------- ----------
Magnetospirillum magn.           FPMGKYGRLA EILR------ -ARGLA-P-- ---------- ---------- ----------
Mesorhizobium loti               FPMSKYPLLM EALR------ -ARGLASP-- ---------- ---------- ----------
Caulobacter crescentus           FPMDKFSRLA ALLE------ -AERVAGP-- ---------- ---------- ----------
Erythrobacter litoralis          FKFDKYFLVM EALR------ -ASGEP---- ---------- ---------- ----------
Emiliania huxleyi                FAMEKYPRLF DRLV------ -QRGVLSPS- ---------- ---------- ----------
Vibrio vulnificus                YPIMKYHHLY QAVC------ -RYQQQHLEW QQA------- ---------- ----------
Vibrio parahaemolyticus          YPIMKYQYLY EEVR------ -R--DVQAEW VQ-------- ---------- ----------
Vibrio cholerae                  YPINKYRLLY EEIV------ -RQREQSEAW QAS------- ---------- ----------
Shewanella oneidensis            FPTTKYAHLY QYLL------ -D--NQLATP TQ-------- ---------- ----------
Idiomarina loihiensis            YPIEKYRLLK DWAV------ -KHGATNNQW HQ-------- ---------- ----------
Solibacter usitatus              FPSQKFRWLR DRLL------ HTRFAAAE-- ---------- ---------- ----------
Schmidtea mediterranea           FPIRKFELVK DLLL------ KEGTLAES-- ---------- ---------- ----------
Drosophila melanogaster          FDAAKGKHIH KLLCAQ---- ----LQLDDG S--------- ---------- ----------
Chlamydomonas reinhardtii        FDAGKFAKVV KALKRD---- ---GVVLGDA Q--------- ---------- ----------
Anopheles gambiae                FDAAKGGNIY RLLKTN---- ---GLIQTDG D--------- ---------- ----------
Arabidopsis thaliana             FDSSKWGRVC KFLVSD---- ---GFLEEKA ---------- ---------- ----------
Aquilegia formosa                ---------- ---------- ---------- ---------- ---------- ----------
Oriza sativa                     FDSSKWGRIC KFLTKE---- ---GHLEKNR ---------- ---------- ----------
Hordeum vulgare                  ---------- ---------- ---------- ---------- ---------- ----------
Picea engelmannii                ---------- ---------- ---------- ---------- ---------- ----------
Homo sapiens                     FDAGKWGKVI NFLKEE---- ---KLLSDSM ---------- ---------- ----------
Mus musculus                     FDAGKWGKVI NFLKEE---- ---KLLSDGM ---------- ---------- ----------
Gallus gallus                    FDAGKWGKVI NFLKEE---- ---KLIGDDL ---------- ---------- ----------
Danio rerio                      FDAGKWGKVI RFLKEE---- ---QFITDEI ---------- ---------- ----------
Strongylocentrotus purp.         FDSGKWGKVY NLLKSK---- ---------- ---------- ---------- ----------
Tribolium castaneum              FDAGKWGNIY KYLKSC---- ---GLVNDET ---------- ---------- ----------
Locusta migratoria               ---------- ---------- ---------- ---------- ---------- ----------
Ciona intestinalis               --------IV WFITDD---- ---KLLQNNQ ---------- ---------- ----------
Dugesia ryukyuensis              --SSKAKAEC QDSENL---- ---INITKNN ---------- ---------- ----------
Solanum tuberosum                FDSSKWGRIC RFLTKE---- ---GIMDQKH ---------- ---------- ----------
Antirrhinum majus                FDSSKWGRIC RFLIVE---- ---GLLDKNC ---------- ---------- ----------
Triticum aestivum                FDSSKWGRIC RFLTKE---- ---GHLEKTR ---------- ---------- ----------
Zea mays                         FESAKWGRIC RYLTRE---- ---GYLDKKQ ---------- ---------- ----------
Heterodera glycines              FDSNKWGNVF RFLIAS---- ---GALVESA ---------- ---------- ----------
Caenorhabditis briggsae          FDSSKWKRVI AHLKDM---- ---N-LITDA T--------- ---------- ----------
Caenorhabditis elegans           ---------- -----M---- ---N-LITDE T--------- ---------- ----------
Tetrahymena thermophila          FDSVKYGRVF NILKEK---- ---GFLQEQG F--------- ---------- ----------
Paramecium tetraurelia           FDSCKYGNVY RQIRQ----- ------QVKG S--------- ---------- ----------
Leptospira interrogans           FPARKYQMVY DLVK------ -RDSKLSNLY ---------- ---------- ----------
```

```
                                        181
Cytophaga hutchinsonii          ---SNLTTPV -RIASEDLLR SQDATYVHKL NTLSLS---- -------PSE IRKTGFE---
Nematostella vectensis          ---HQLFSPA -FCAYEDLNT VHDALYLQQL FDLKIS---- -------PRE QRKTGFV---
Psychrobacter cryohalolentis    ---NNFFAPT -RLSEDEILT THTADYWYQL KTQTLP---- -------RKE ARAIGFE---
Phaeodactylum tricornutum       ---YEFRVSP -LSSIDELTT THDPAYVQRF LTGDQD---- -------ERE LRNVGFP---
Thalassiosira pseudonana        ---------- ---------- ---------- ---------- ---------- ----------
Cyanidioschyzon merolae         ---VPFLVSP -LAELDDILL VHSKDYVDRF IKGKLS---- -------DIE VRRIGFP---
Ostreococcus tauri              ---IELRASP -LVDIEDLEA AHCGEYVRKV LTRTLS---- -------EQE VRTIGFP---
Ralstonia metallidurans         ----ELQEAP -RADDATLAL AHTAAYIEDV STGQLD---- -------AAR QREIGFP---
Ralstonia solanacearum          ----VMHEAP -RAGDDALLL AHAPDYVSAV GAGRLD---- -------PAR QREIGFP---
Rubrivivax gelatinosus          ----RLVEAP -AASDGELAL AHEPAWVNAV LDGTLS---- -------ATQ QREIGFP---
Polaromonas sp                  ----QMAQAP -AASDGELAL VHTPAYIEAI THGSLP---- -------PAA QREIGFP---
Pirellula sp                    ----TLIVPQ -AATDEQLLH CHTPDYVQRV QSGTLT---- -------KQE IRRIGFP---
Rhodopirellula baltica          ----TLIVPQ -AATDEQLLH CHTPDYVQRV QSGTLT---- -------KQE IRRIGFP---
Chloroflexus aurantiacus        ----RLHVPE -PASITELCR AHDANYVERV INGHLT---- -------SAE IRRIGFP---
Dechloromonas aromatica         ----DFQLPH -AATDEELGR AHDLDYIQAI SIGELS---- -------EAA QKAIGFP---
Chromobacterium violaceum       -----METAP -AATRGELIH AHHPDYVDAV LNGTLD---- -------ARA QREIGLP---
Rubrobacter xylanophilus        ----GLREPR -RAKDEDLLR AHSPSYLRRV VSGSLG---- -------KQE LRRLGFP---
Thermus thermophilus            ------LPAP -EVPREALFL AHEASYLEKL FGEGLT---- -------REE SLRLGLP---
Deinococcus radiodurans         ------LPTP -QLRWADAGR AHDPLWLRRW RRGEVD---- -------RRE QREFGLP---
Pseudomonas putida              QALLRPDI-- --CPNDILAL AHDRSYIERY MNGE------ -----LSRED QRRLGLPWS-
Pseudomonas fluorescens         EDLLRPEL-- --CPADILAL AHDRSYIERY MSGE------ -----LSRED QRRLGLPWN-
Pseudomonas syringae            VQLMRPEL-- --CPADILAL AHDPSYISRY LSGD------ -----LSRED QRRLGLPWS-
Azotobacter vinelandii          AELRRPEP-- --CPTDILAL AHDPAYIERY CSGA------ -----LSREE LRRLGLPWT-
Phaeodactylum tricornutum       SSLPRPLVRQ -ELDFFRPLD FGDVPRSWLS GPIDSVFVDR FLNAQLSHEE CRVIGFREQT
Thalassiosira pseudonana        ---------- ---------- ---------- ---------- ---------- ----------
Ostreococcus tauri              SSSNAFSPSR -RPTFEELAA AHSEEWTRTA TSSEG----- -----PDAKR LREIGLPWSD
Chlamydomonas reinhardtii       ---------- ---------V VHDPDYLALF SSGR------ -----LDEER VRRIGP----
Nostoc sp                       ----QFYTPT -LPPLELIEL VHTPDYVRSY CEGTLD---- -------TKA QRRIGLP---
Anabaena variabilis             ----QFYTPI -LPHPELIEL VHTPDYVRSY CEGTLD---- -------TKA QRRIGLP---
Nostoc punctiforme              ----QFHTPE -RPPPELIEL VHTPSYVQAY CEGTLD---- -------PKL QRRIGLP---
Trichodesmium  erythraeum       ----YFLTPN -FPPLEWIEL IHHPNYIKKY CQGTLD---- -------PKA QRRIGLP---
Crocosphaera watsonii           ----DIYTPK -LPENSLLEL VHCPDYITGY CQGTLT---- -------DKA QRRIGLP---
Synechococcus sp                ----QVYQPQ -LPDRAWLEL VHEPDYVTAY CQGTLT---- -------PKA QRRIGLP---
Synechococcus elongatus         ----QFHQPD -RPPLEWLTS VHSLDYIQAY SQGTLD---- -------ERS LRRIGLP---
Gloeobacter violaceus           ----QFWEPE -RAGWEWLTL VHAPRYVADF CAGILS---- -------AQE FRRIGLP---
Nematostella vectensis          ---------- ---------- ---------- ---------- ---------- ----------
Locusta migratoria              ---KQVMNPK -QVSRNTACC VHTAEYVDKF FNGKTS---- -------EEE QRVTGFV---
Callinectes sapidus             ---KQVILPQ -KVSRECASA VHSQVYVDKF FDGETS---- -------TAE QRVTGFV---
Platynereis dumerilii           ---KQVAEPD -EVSSQVAGL VHTPEYVEKF FTGKTS---- -------EKE QRRTGFK---
Strongylocentrotus purp.        AKEQRMSSPV -HVTKDELIR VHTEEYIEKF FEGKTS---- -------AKE QRVTGFT---
Takifugu rubripes               -------VPE -IASSDLLSC VHTEEYLNNF MNGRIN---- -------EQE QRRTGFP---
Oryzias latipes                 ------WVPE -IASRDLLSC VHTEDYLNKF LSGKTS---- -------EQE QRRTGF----
Gasterosteus aculeatus          ------WLPE -IASEDLLRC VHTEEYLNNF IGGKTN---- -------EQE QRRTGFP---
Pimephales promelas             ---------- ---------- ---------- ---------- ---------- ----------
Magnetospirillum magn.          ---DGFVTPE -PAGAALLSG AHEAAYVEAV LAFQVP---- -------RAV ERAIGLP---
Mesorhizobium loti              ---DALNTTE -PAPASWLKL AHAADYVDQV ISCSVP---- -------EKI EREIGFP---
Caulobacter crescentus          ---DGFARPE -PVDVETLCL AHSEDYVRGV IELSLP---- -------PDI VRRIGMP---
Erythrobacter litoralis         ---ITEHAPE -PMPRQWLEA VHCPEYVEQV FTASVP---- -------REK ERRIGFP---
Emiliania huxleyi               ---EVCEPAD -PAGNEWLHR AHSAEYVAGY CDGTLA---- -------PNA MRRIGLP---
Vibrio vulnificus               ---FAFYQPE -ALSIEAIKQ VHQQEYVDLL TTGLLP---- -------AAK MRRIGFP---
Vibrio parahaemolyticus         -----FFEPQ -ALSIEAIKR VHDADYVDLL AQGNMP---- -------AAK MRRIGFP---
Vibrio cholerae                 ---FEFHTPI -AAELSRITR LHDPDYVQAL LEGTLS---- -------AAK MRRIGFP---
Shewanella oneidensis           -----FHTPT -PMTAEEIMQ VHHRDYVEQF IDGTLA---- -------TSA LRRIGFP---
Idiomarina loihiensis           --------PE -PLSWEQVAR THCPGYLSQL RQNSMD---- -------KSS WRRIGFP---
Solibacter usitatus             ----DFVTPE -SATDDDVRL VHDPEYVAKL RGGTLS---- -------YQD ILRLEIP---
Schmidtea mediterranea          ----EIIAPE -PAKKEDLLL VHTEDYISRL VDGTLT---- -------AKE IRKLGLP---
Drosophila melanogaster         -----FYEPT -ELTKDQLRR IHTREYLKSL ---RWSMN-- ---VACIAEV PLMAFVPNRY
Chlamydomonas reinhardtii       -----VCAYV -LVWKIDLLV VHTENYLNSL ---KWSAT-- ---VARITEV PPMAFVPNFL
Anopheles gambiae               -----VYAPN -EITLEELLA VHTQRYIDSL ---KWSLN-- ---VAKIAEI PPLLFVPNCF
Arabidopsis thaliana            -----IVEPL -EASKIDLLV VHSENYLNSL ---KSSAT-- ---VARITEV APVAFFPNFL
Aquilegia formosa               ---------- ---------- ---------- -----SLK-- ---VSIIMEV PPVALLPNCV
Oriza sativa                    -----VVEPL -EATKDDLLV VHSESYLNSL ---KSSLK-- ---VASIVEL PPVAFIPNWL
Hordeum vulgare                 ---------- ---------- ---------- ---------- ---VAAIVEV PPLTLIPNWL
Picea engelmannii               ---------- ---------- ---------- ---KSSSN-- ---IAFIIEV PPVALLPNFM
Homo sapiens                    -----LVEAR -EASEEDLLV VHTRRYLNEL ---KWSFA-- ---VATITEI PPVIFLPNFL
Mus musculus                    -----LVEAR -EASEEDLLV VHTRRYLNEL ---KWSFV-- ---VATITEI PPVIFLPNFL
Gallus gallus                   -----IVQAR -EATDEDLLV VHTRRYLNKL ---KWSFV-- ---VATITEI PPVLFLPNFL
Danio rerio                     -----IVLAR -EASEADLLV VHTARYLNRL ---KWSLV-- ---VATITEI PPLLFLPNFL
Strongylocentrotus purp.        ---------- ---------- ---------- ---QWSIS-- ---VAGITEI PPVALLPNYI
Tribolium castaneum             -----LSVPN -EATTEDLLT VHTKKYLKSL ---K-SFN-- ---VALIAEV LPLCLVPNYL
Locusta migratoria              ---------- ---------G LQAARYLQSL ---KFSFN-- ---VAAVAEI PLLALVPNFF
Ciona intestinalis              -----HFQPL -EPTERDLLI AHSKEYLSSL KVSQWSAN-- ---VARITEV PPVALLPNFI
Dugesia ryukyuensis             -----ITEAV -EARDEELRI GHSSEYLNSL ---KKSLN-- ---VAKIVEV GLVAMIPNRF
Solanum tuberosum               -----VVEPV -EATKDDLLV VHSESYLKSL ---NSSLN-- ---VSMIVEV PPVAMLPNCL
Antirrhinum majus               -----IVEPL -EAKKDDLLV VHSEAYLNSL ---KSSLN-- ---VATIVEV PPVALLPNCL
Triticum aestivum               -----VVEPL -EASKEDLLV VHTEAYLNSL ---RSSFR-- ---VAAIVEV PPLTLMPNWL
Zea mays                        -----MVEPL -EACKEDLLV VHTEAYLNSL ---KCSFR-- ---VSSIVEV PPVSLVPNWI
Heterodera glycines             -----VLRPI -EAKKEHLLL VHSKKYLRSL ---NGRFT-- ---LTRILEV GLVLLFPICL
Caenorhabditis briggsae         -----TVEPS -LPTFEELTR VHDRKYLKSV ---RNPLK-- ---AAQIVEI PLVGCLPPCV
Caenorhabditis elegans          -----LVEPN -LPTFEELTR VHDRKYLKSV ---RNPIK-- ---AAQIVEI PFVGCLPPCI
Tetrahymena thermophila         -----YKPKE KVGRGLMLHL GMSPLYLLYL ---NYAAY-- ---VSKCIEI PLF-FLPASF
Paramecium tetraurelia          -----HFTPK MLSRGTFLYL GMSKWYLLKM ---CYSAY-- ---ASTLIEL PVF-FLPGAF
Leptospira interrogans          -----IYKPD -LAKTKDLSL VHTQEFLDDF FSLNIT---- -------ERT QYS-ELP---
```

```
                              241
Cytophaga hutchinsonii        LTAELVYREE VIMQG-TIDS ALHALKHG-I GMN------- IAGGTHHAYK EH-----GEG
Nematostella vectensis        HSSSLIERES RIMEG-TRKC AEFALSHG-A AMN------- IAGGTHHAYS NR-----GEG
Psychrobacter cryohalolentis  MTPELVERGR YIAHA-TYEC ALYAQQYG-V AMN------- VAGGTHHAFS DH-----GEG
Phaeodactylum tricornutum     WSQSNVDRSL SSTGG-TVAA ACAVVQARLR DPYGLHWGAH VAGGTHHAFY DR-----GEG
Thalassiosira pseudonana      ---------- --VGG-TVAA ACAAWEDTTI CIY------- VAGGTHHAFS DY-----GEG
Cyanidioschyzon merolae       WSTALVQRTL ASVGG-TVAC MRDVVEGR-S RCAAQ----- IAGGTHHAFP DH-----GEG
Ostreococcus tauri            MGEQNVTRSL ASTGG-TVAC AREVLAGFGA RAAAQ----- LAGGTHHAYR DR-----GEG
Ralstonia metallidurans       WSHEMVERSR RSAGA-TIAA CRVAL----- ---EQGIAAN LAGGTHHAYA DK-----GAG
Ralstonia solanacearum        WSPEMVERSR RSAGA-TMAA CEAAM----- ---ADGIAVN LAGGTHHAYA DK-----GGG
Rubrivivax gelatinosus        WSEAMVERSR RSVGA-TIAA ARRAL----- ---DEGVAAN LAGGTHHASA DK-----GSG
Polaromonas sp                WSPGMAERAR RSAGA-TVAA ARVALGTG-- -TRPQGVAAN MAGGTHHAYA HK-----GSG
Pirellula sp                  WSAKMVERSR RSTGA-TISA ARAAL----- ---DEGISAN LAGGTHHAFA GE-----GEG
Rhodopirellula baltica        WSAKMVERSR RSTGA-TISA ARAAL----- ---DEGISAN LAGGTHHAFA GE-----GEG
Chloroflexus aurantiacus      WSPQMVERSR RSAGA-TIAA CRVAL----- ---SEGVGVN LAGGTHHAFA DA-----GAG
Dechloromonas aromatica       WSQGMVERSR RSAGA-TICA CRAAL----- ---AEDVSAN LAGGTHHAFR DH-----GEG
Chromobacterium violaceum     WSPELAERSR RSVGA-TVAA SRSAL----- ---LEGCGVN LAGGTHHAGR ER-----GSG
Rubrobacter xylanophilus      WSERLVERSR RAAGG-TIDA CLAAL----- ---EEGIAAN LAGGTHHAFA DR-----GEG
Thermus thermophilus          FSQALLRRAL HAAGG-TLAA ALDAL----- ---KTGLGLN LSGGTHHAFP GR-----AEG
Deinococcus radiodurans       WSPEVVTRAL RAAGG-SLAA LHDAQ----- ---STGWGAN LAGGTHHAFH DR-----AEG
Pseudomonas putida            --EALARRTV RAVGG-SLLT AEMAL----- ---QHGIACH LAGGTHHAHY DH-----PAG
Pseudomonas fluorescens       --EALARRTV RAVGG-SILA AEKAL----- ---EHGLACH LAGGTHHAHY DY-----PAG
Pseudomonas syringae          --EALARRTI RAVGG-SLLT AEQAL----- ---KHGLACH LAGGTHHAHY DY-----PAG
Azotobacter vinelandii        --PALARRTV LAVGG-SLLA AELAL----- ---EHGLACH LAGGTHHAHH DH-----PSG
Phaeodactylum tricornutum     GRTELIRRTV LEVAG-TVLT SQLAC----- ---RYGIAAH VAGGTHHAHV TG-----GAG
Thalassiosira pseudonana      ----LIERTV LEVAG-TVLT AQLAM----- ---KYGLACH LAGGTHHAES CR-----GKG
Ostreococcus tauri            ---VLVERTL MEVSG-TMLT VEMAL----- ---ECGLAVN TAGGTHHAKG TR-----GGG
Chlamydomonas reinhardtii     ----LVRRTK AEVAG-TLLT ARLAL----- ---SGGLAVN TAGGTHHAFP SH-----GSG
Nostoc sp                     WSPALANRTC IAVGG-TILT AQLAL----- ---SQGLACN TAGGTHHAFP SY-----GSG
Anabaena variabilis           WSPALANRTC IAVGG-TILT AQLAL----- ---NQGLACN TAGGTHHAFP SY-----GSG
Nostoc punctiforme            WSPALANRTC VAVGG-TILT AKLAL----- ---SHGLACN TAGGTHHAFP SY-----GSG
Trichodesmium  erythraeum     WSQALANRTC IAVGG-TILT AQLAL----- ---KHGLACN TAGGTHHAFP SY-----GSG
Crocosphaera watsonii         WSEALVKRTC IAVGG-TILT AKLAL----- ---KFGLACN TAGGTHHAFP NY-----GSG
Synechococcus sp              WSAGVVQRTL TAVGG-TILT AQLAL----- ---EHGLACN TAGGTHHAFP GY-----GSG
Synechococcus elongatus       WSPALVERTW IAVGG-TILT ARLAL----- ---QQGLACN LAGGTHHAFP DY-----GSG
Gloeobacter violaceus         WSPALVERTC TAVGG-TILT ARLAL----- ---RHGLACN TCGGTHHAFP DF-----GSG
Nematostella vectensis        ---------R FSPGG-TILT ARIAL----- ---ECGLACS TGGGTHHAFP SH-----GSG
Locusta migratoria            WSPGLASRVR YETGG-TLLT AALAF----- ---ERGLACS TAGGTHHAFP DR-----GSG
Callinectes sapidus           WTPGLASRVR YETGG-TCLG AKLSL----- ---ECGLACS TGGGTHHAFP DH-----GSG
Platynereis dumerilii         WNKGLLRRCR LEAGG-TVLG CHLAK----- ---ERGLACS TGGGTHHAFP SY-----GSG
Strongylocentrotus purp.      WSEGLVSRCR YETGG-TILA AELAL----- ---GRGLVCN TGGGTHHAFP EH-----GAG
Takifugu rubripes             WSEGIVTRCR YETGG-TLLA AELAL----- ---QRGLACS TAGGTHHAFP GF-----GSG
Oryzias latipes               WSEGIVSRCR YETGG-TVLA AEAAL----- ---QRGLACS TAGGTHHAFP TY-----TR-
Gasterosteus aculeatus        WSEGIVRRCR YETGGGTVLA AEAAL----- ---QRGLACS TAGGTHHAFP SY-----GSG
Pimephales promelas           ---------- ---------- ---------- -----CLLND LAVTAKHLMG ET-----AS-
Magnetospirillum magn.        VTEAVAARSR ASAGG-TLCA ARLAL----- ---EHGLAGS TAGGSHHARR AG-----GAG
Mesorhizobium loti            VGPRVSLRAQ LATGG-TILA ARLAL----- ---RHGIACN TAGGSHHARR AQ-----GAG
Caulobacter crescentus        NTESVATRAR AATGG-TLLA ARLAL----- ---ERGIACN TAGGSHHAAA DA-----GAG
Erythrobacter litoralis       VTAHIASRVR HTNGG-TWLA AQLAM----- ---EHGYAAN SAAGSHHALF DT-----GAG
Emiliania huxleyi             WSPAMVRAVR LEVAG-TLLA ARLAL----- ---STGIACN LGGGTHHAQR AA-----GSG
Vibrio vulnificus             WSEKLIERTL TSTAG-TVLT AEKAL----- ---QHGVAIH LSGGYHHAHF DY-----GSG
Vibrio parahaemolyticus       WSEALITRTL TSAAG-TLLT AEKAL----- ---EHGIALH LSGGYHHAHK DF-----GSG
Vibrio cholerae               WSKPLIERTL YSVGG-TCLT VEQAL----- ---QSGVAIH LSGGYHHAHA DF-----GSG
Shewanella oneidensis         WSEALVERTL HSLAG-TSLT AALAL----- ---QTGIALH LTGGYHHAHY EF-----GSG
Idiomarina loihiensis         WSEQLLYRTL TSAGG-TLLT TELAL----- ---TKGVAIH FSGGYHHAHK DW-----GSG
Solibacter usitatus           YSRQMVEAFW LAAGG-SILA ARLAL----- ---QDGIGFN IGGGFHHAFP GH-----GEG
Schmidtea mediterranea        WSESLVRRSF HAISG-TINA ARFAL----- ---QDGISSN LAGGTHHAFP DR-----GEG
Drosophila melanogaster       IQRSYLRPMR FQAAG-SILA GKLALDY--- -----GWAIN LGGGFHHCCS YR-----GGG
Chlamydomonas reinhardtii     VQQKVLRPGR MHVGG-TMLA LGLALER--- -----GWAVN IGGGMHHASA DR-----GMG
Anopheles gambiae             VQRSYLRPMR YQTGG-SLLA ARAALESG-- ----LGWAIN LGGGFHHCSA DR-----GGG
Arabidopsis thaliana          VQQKVLYPFR KQVGG-TILA AKLATER--- -----GWAIN IGGGFHHCTA ER-----GGG
Aquilegia formosa             VQQKALYPFR KQVGG-TILA AKLAKER--- -----GWAIN VGGGFHHCCA EK-----GGG
Oriza sativa                  VQQKLLYPFR KQVGG-SILS AKLALER--- -----GWAIN VGGGFHHCSA EQ-----GGG
Hordeum vulgare               VQQRLLYPFR KQVGG-SILS AKLALER--- -----GWAIN VGGGFHHCSA EE-----GGG
Picea engelmannii             CGKRVLQPFC KQVGG-TVLA GKLAKER--- -----GWAIN LGGGFHHCSK NK-----GGG
Homo sapiens                  VQRKVLRPLR TQTGG-TIMA GKLAVER--- -----GWAIN VGGGFHHCSS DR-----GGG
Mus musculus                  VQRKVLRPLR TQTGG-TIMA GKLAVER--- -----GWAIN VGGGFHHCSS DR-----GGG
Gallus gallus                 VQRKVLKPLR TQTGG-TIMA GKLAVDR--- -----GWAIN VGGGFHHCSS DK-----GGG
Danio rerio                   VQRKVLRPLR TQTGG-TIMA GKLAIDR--- -----GWAIN VGGGFHHCSS DK-----GGG
Strongylocentrotus purp.      VQKKVLRPLR LQTGG-SLQA AKLAMER--- -----GWAIN IGGGFHHCSS KQ-----GGG
Tribolium castaneum           VQRGYLRPMR FQTGG-SVLA GKLALER--- -----GWAIN IGGGFHHCCG SK-----GGG
Locusta migratoria            VQSHYLRPMR YQVGG-SILA GKLALER--- -----GWAIN IGGGFHHCCG QK-----GGG
Ciona intestinalis            VQRKVLLPLR LQTGG-SVLA GKLAIER--- -----GW-IN IGGGFHHCSG DR-----GGG
Dugesia ryukyuensis           VQSRLLSKFR YQVGG-TIQA AKLALKR--- -----SWAIN IGGGFHHASE DA-----GGG
Solanum tuberosum             VQKKVLHPFR KQVGG-TILA AKLAKER--- -----GWAIN VGCGFHHCSS EK-----GGG
Antirrhinum majus             VDKHVLYPFR KQVGG-TILA AKLAKER--- -----GWAIN VGGGFHHCSA EK-----GGG
Triticum aestivum             VQQRLLYPFR KQVGG-SILS AKLALER--- -----GWAIN VGGGFHHCSA EE-----GGG
Zea mays                      VHRKLLHPFR KQVGG-SILS AKLAFER--- -----GWAIN VGGGFHHCSA DE-----GGG
Heterodera glycines           IDRRILRPMR IQTGG-TVLA ARVSLIR--- -----GWAIN IGGGFHHASE GK-----GGG
Caenorhabditis briggsae       IET------- ---GG-SVLA ANLALKH--- -----GWAIN VGGGFHHASY SD-----GGG
Caenorhabditis elegans        IESKLLHPLR LQAGG-TVLA ANLALKH--- -----GWAIN VGGGFHHASH SG-----GGG
Tetrahymena thermophila       LRWKVLDPMM FSTQG-SIDA AVLSLKR--- -----GWSIN LSGGYHHACI NRNKKQSGGG
Paramecium tetraurelia        LRSCLLDSML LATSG-SIQA AKLALEK--- -----GWAIN LSGGYHHASL NR-----GGG
Leptospira interrogans        LTKQIVHSFV LAVGG-TILS MELAQ----- ---KYKFVYH IGGGFHHSMP DR-----AEG
```

```
                                301
Cytophaga hutchinsonii          FCLYNDIILA ADYLLAN--K ---------- -LAHQVLVVD LDVHQGNGTA KMAEGNT---
Nematostella vectensis          FCLMNDQAIA AKWLLKN--K ---------- -LTTQVLIID LDVHQGNGTA QVFANTP---
Psychrobacter cryohalolentis    FCVFNDVCIA SNLLLNR--G ---------- -QAQKILVVD LDVHQGNGNA SIMANEP---
Phaeodactylum tricornutum       FCVFSDMAVA ANVVMKRYPD ---------- -IVRRILFLD LDVHQGNGNA LLFRDND---
Thalassiosira pseudonana        FCIFSDIAVA ANVLLQKYSH RTQQHHTQKR SGIRRILIID LDVHQGNGNA KLFDGNS---
Cyanidioschyzon merolae         FCVFNDIAIA ARVALRD--Y A--------- -QVRRILVLD LDVHQGNGVA AIFQGDS---
Ostreococcus tauri              FCVFNDIGTA IRVVQRDELL P--------- -RDRKILVID LDVHQGNGTA KMFEHDQ---
Ralstonia metallidurans         FCVFNDAAIA ARRLQR-DG- ---------- -SVRRVAVID LDVHQGNGTA SILRDDP---
Ralstonia solanacearum          FCVFNDAAIA SRWMQRRPGR ---------T PEHFPVAIVD LDVHQGNGTA SILRDDA---
Rubrivivax gelatinosus          YCVFNDIAVA ARLMQAEW-- ------HRAR RTLLRVAVID LDVHQGNGTA AIFRDDP---
Polaromonas sp                  FCVFNDSAVT ARLMQAEWGR R-----HRPD RKPLQVAVID LDVHQGNGTA HIFANDP---
Pirellula sp                    YCVFNDAAVA IRTLQSEG-- ---------- -LIQRAAIID LDVHQGNGTA SILKDVP---
Rhodopirellula baltica          YCVFNDAAVA IRTLQSEG-- ---------- -LIQRAAIID LDVHQGNGTA SILKDVP---
Chloroflexus aurantiacus        YCVFNDAAVA ARAMQSEG-- ---------- -RVQRIAIID CDVHQGDGTA AIFTGDP---
Dechloromonas aromatica         FCIFNDAAVA ARTMQAEG-- ---------- -RAKRVLIVD CDVHQGNGTA SILRGDD---
Chromobacterium violaceum       FCMFNDIAVA SMLLLAEA-- ---------- -RVRRVLIVD LDVHQGDGTA AIAADEP---
Rubrobacter xylanophilus        FCVFNDAAVA IRAVQAAG-- ---------- -LVERAAVID TDVHQGDGTA AIFAADG---
Thermus thermophilus            YSLFNDVAVA IFWLRAKEG- ---------- -FSGRVLVVD LDAHQGNGTA FFFREDP---
Deinococcus radiodurans         FCLVNDAAIL TR-IALDRG- ---------- -LARRVATLD LDVHQGNGTA SLLTPEMAAG
Pseudomonas putida              FCIFNDLAVI SRYLLEAGR- ---------- --VHRVLIFD CDVHQGDGTA RILHDTP---
Pseudomonas fluorescens         FCIFNDLAII SHYLLQSGR- ---------- --VNRVLIFD CDVHQGDGTA RILHNTP---
Pseudomonas syringae            FCIFNDLAVI SQYLLQSGR- ---------- --VDKVLIFD CDVHQGDGTA RILADTE---
Azotobacter vinelandii          FCIFNDLAVV SRYLLASGR- ---------- --VGRVLIFD CDVHQGDGTA RILEDTP---
Phaeodactylum tricornutum       YTIFNDLAVA THVVTATEP- ---------- -SVERVLVID CDVHQGDGTA RFGAAPDGPL
Thalassiosira pseudonana        FTILNDLAVV ARLMTWNEGD ---------- -EVERVLVVD CDVHQGDGTA TFHTDQTSPL
Ostreococcus tauri              FCILNDLATA SLAVLNSGR- ---------- --LSRVMIVD LDVHQGDGTA EILENEW---
Chlamydomonas reinhardtii       YCILNDLAVR VCVRVTVYV- ---------- --CERVCTCD CNVQ-GDGTA FIFRDRP---
Nostoc sp                       FCIFNDLAIA SRVLQQ-QQ- ---------- -IVQKILIVD LDVHQGDGTA FIFQDDD---
Anabaena variabilis             FCIFNDIAIA SRVLQQ-QQ- ---------- -LVKKILIVD LDVHQGDGTA FIFQDDD---
Nostoc punctiforme              FCIFNDLAIA CRVLQK-FG- ---------- -LVQKILIVD LDVHQGDGTA FIFQDDD---
Trichodesmium  erythraeum       FCIFNDLAIA TRVMQK-LG- ---------- -LVEKVLIVD LDVHQGDGTA WIFQDDP---
Crocosphaera watsonii           FCIFNDLAIA TCVLQQ-LK- ---------- -LVQKVLIID LDVHQGDGTA KIFENDE---
Synechococcus sp                FCILNDLAIA TRTIQQ-RG- ---------- -LAQRILIVD LDVHQGDGTA FIFQDDP---
Synechococcus elongatus         FCIFNDCAIA ARLLLQ-ER- ---------- -LVERILIVD LDVHQGDGTA WIFQDDP---
Gloeobacter violaceus           FCIFNDLAVS ARVLLA-EG- ---------- -QVRRVLIVD LDVHQGDGTA WIFADEP---
Nematostella vectensis          FCIFNDLAIT ASYLLD-NN- ---------- -LVTRVMIVD LDVHQGDGTA SIFQNEP---
Locusta migratoria              FCLINDLAVT AKWFLD-QQ- ---------- -RISRVLIVD LDVHQ----- ----------
Callinectes sapidus             YCLINDLAVA AYHLLQ-SG- ---------- -MVEKVLIVD LDVHQGDGTA AIFQNEP---
Platynereis dumerilii           YCLLNDLAIA AENSVA-TG- ---------- -VAERVLIVD LDVHQGDGTA NIFEDSD---
Strongylocentrotus purp.        FCLLNDMAVA ASLMVH-RG- ---------- -KVDRVLIID LDVHQGDATA LIFQEDP---
Takifugu rubripes               FCLLNDLAVA AKYVS--AS- ---------- -LKRKVLIVD LDVHQGDGTA FIFKEEP---
Oryzias latipes                 ---------- ---------- ---------- ---------- -----GDGTA FIFKEEP---
Gasterosteus aculeatus          FCLLNDLAVA ARYLMGNSS- ---------- -TKRKVLIVD LDVHQGDGTA FIFQEEP---
Pimephales promelas             ---------- ---------- ---------- --KRKILIVD LDVHQGDGTA FIFKDEP---
Magnetospirillum magn.          FCVFNDVAVA ALALRR-EG- ---------- -RIARALVID LDVHQGDGTA DCLAREP---
Mesorhizobium loti              FCTFNDVAVA SLVLLD-EG- ---------- -AAQNILVVD LDVHQGDGTA DILSDEP---
Caulobacter crescentus          FCVFNDVAVA ARRLLA-EG- ---------- -AIGKALVVD LDVHQGDGTA RIFENDP---
Erythrobacter litoralis         YCVFNDLAVA ANRLIA-EG- ---------- -DAKRVLVVD LDVHQGDGTA SLTALRD---
Emiliania huxleyi               FNPFNDLAVA SLALLA-EG- ---------- -AARRVLVVD LDVHQGDGTA AILSEEP---
Vibrio vulnificus               FCLFNDLVMA AHKALEHGS- ---------- --VDKVLIID SDVHHGDGTA TLCQRRD---
Vibrio parahaemolyticus         FCLFNDLVIA AKHMLDNEH- ---------- --VDKILIID SDVHHGDGTA TLCQEEP---
Vibrio cholerae                 FCLFNDLAIA AHFALSLPS- ---------- --VDKVLIID SDVHHGDGTA TLCAERD---
Shewanella oneidensis           YCIFNDLIIA ARKLIIEQQ- ---------- --LHKILIFD CDVHQGDGTA TLSQLHQ---
Idiomarina loihiensis           FCLLNDLAIA CNEILVRHP- ---------- --KLKIVVLD TDVHQGDGTA TLFENDN---
Solibacter usitatus             FCAINDVAIA VRRLQADR-- ---------- -LIKRAMVVD CDVHHGNGTA AIFTDDQ---
Schmidtea mediterranea          FCVLNDVAVA IRVLQREK-- ---------- -LAQNFLIVD CDVHQGNGTA FIFQNSP---
Drosophila melanogaster         FCPYADISLL IVRLFEQEPF ---------- -RVRRIMIVD LDAHQGNGHE RDFNNVAA--
Chlamydomonas reinhardtii       WCPFDDIMLG ---------- ---------- --VRKVLVID LDAHQGNGVE RLGD--GQ--
Anopheles gambiae               FCPYADITLA VKMLQSSGK- ---------- -GIERILIVD LDAHQGNGYE RDLMEDRR--
Arabidopsis thaliana            FCAFADISLC IHFAFLRLR- ---------- --ISRVMIID LDAHQGNGHE TDLGDDNR--
Aquilegia formosa               FCAYADISIC IHFAFIQLN- ---------- --ISRVMIID LDAHQGNGHE MDFSKDGR--
Oriza sativa                    FCAYADISLC IQFAFVRLN- ---------- --ISRVMIID LDAHQGNGHE KDFANDGR--
Hordeum vulgare                 FCAYADISLC IQFAFVRLN- ---------- --ISRVMIID LDAHQGNGYE KDFANDGR--
Picea engelmannii               FCVYADISLC IQFAFAQLG- ---------- --ISKVMIID LDAHQGNGHE MDFAHDGR--
Homo sapiens                    FCAYADITLA IKFLFERVE- ---------- -GISRATIID LDAHQGNGHE RDFMDDKR--
Mus musculus                    FCAYADITLA IKFLFERVE- ---------- -GISRATIID LDAHQGNGHE RDFMGDKR--
Gallus gallus                   FCAYADITLA IKFLFERVP- ---------- -GVSKATIID LDAHQGNGHE RDFMNDHR--
Danio rerio                     FCAYADITLA IKFLFERVE- ---------- -GVASATIID LDAHQGNGHE RDFLEDRR--
Strongylocentrotus purp.        FCAYADITLA LRFLFHQGT- ---------- --IKKAMILD LDAHQGNGHE RDFMQDKES-
Tribolium castaneum             FCVYADITLL IHFVFNHHPR S--------- --VQNVMIVD LDAHQGNGYQ RDFKDNPN--
Locusta migratoria              FCPYADITLL IRFLFRHG-- R--------- --ARTAMIVD LDAHQGNGHE RDFSGDNS--
Ciona intestinalis              FCAYADITLL IKLQFVFAKF D--------- -AIKRVMVVD LDAHQGNGYA RDFMFDDR--
Dugesia ryukyuensis             FCVYADISIA IKSIQQENP- ---------- --DYKFMIID LDAHQGNGHE RDFTNDKN--
Solanum tuberosum               FCVYADISLC IHFAFVRLN- ---------- --ISRVMIID LDAHQGNGHE MDFSDDKR--
Antirrhinum majus               FCVYADISLC IHFAFTRLN- ---------- --ISRVMIID LDAHQGNGHE KDFCD-----
Triticum aestivum               FCAYADISLS IQFAFVRLD- ---------- --ISRVMIID LDAHQGNGHE EDFANDGK--
Zea mays                        FCAYADISLC IQFAFVRLN- ---------- --ISSVLIID LDAHQGNGHE KDFANDGR--
Heterodera glycines             FCVYADVTLA IKLLFANEL- ---------- --IKSAMIVD VDAHQGNGHE TDFASDSR--
Caenorhabditis briggsae         FCFYADITMA ICDLFDKKA- ---------- --ITNAIVVD VDAHQGNGHA RDFANN-EN-
Caenorhabditis elegans          FCFYADITMA IFDLFDKKA- ---------- --IANAIVVD LDAHQGNGHA RDFADN-PN-
Tetrahymena thermophila         FCIYPDICFA IEYLRKCFG- ---------- --IKRCMIVD LDAHQGNGHE RDFIDDKEN-
Paramecium tetraurelia          FCIYPDITLV VNYLKRCCN- ---------- --LKKIVIVD LDAHQGNGYE RDFLND-SS-
Leptospira interrogans          FCYLNDAAIA SKLYQKEYP- ---------- --DKKILFID LDLHQGNGNS FIFQNDP---
```

```
                                 361
Cytophaga hutchinsonii          --QIFTFSMH GASNYPAHK- --EQSDLDIG LPDKTGDKAY LNLLE----E NLSRLI--DT
Nematostella vectensis          --EVFTFSMH GKNNYPLKK- --EMSDCDVE LEDGIGDTAY LKLLE----R HLSETL--ER
Psychrobacter cryohalolentis    --RVFIFSMH GAKNYPFRK- --QVSDLDIE LDNDTGDEQY LQILE----D TLPRLI--SD
Phaeodactylum tricornutum       --SVFTFSLH CSANYFSEK- --QNSDLDIE LPPECSDETY LLTLK----H WLNRIER-EA
Thalassiosira pseudonana        --DVWTFSMH CQGNYFSKK- --ETSDLDIE LPIGCGDETY ISTLS----H CEDDTK--VK
Cyanidioschyzon merolae         --RVFTCSFH GHGNYPFRK- --QKSDLDIE FEDNTDDTDY LQILE----V WLPRIM--AK
Ostreococcus tauri              --QVVTFSMH GEKNYPIKTR --ERSTHDVG LPDDANDEMF LEQLD----H WLPRLW--QE
Ralstonia metallidurans         --TIFTLSLH GEKNYPFRK- --EASDLDVG LPDGCDDGTY AVALA-SALE TLFARF----
Ralstonia solanacearum          --SVFTLSEH GEKNYPFRK- --EASDLDVG LPDGCGDAAY LEALA-GALD TLAARF----
Rubrivivax gelatinosus          --TVFTLSLH GEKNFPFRK- --EASDLDVE LPDGCRDAAY LAALD-GALA TLWARHS-DA
Polaromonas sp                  --SVFTLSLH GQKNFPFRK- --EASDLDVE LPDGCTDAPY LQALE-HALD ELDRRF--D-
Pirellula sp                    --SVFTCSVH GVKNFPLRK- --MPSDLDVS LPDGTGDDDY CDALR-SVLA KLEKHQS-ES
Rhodopirellula baltica          --SVFTCSVH GVKNFPLRK- --MPSDLDVS LPDGTGDDDY CDALR-SVLA KLEKHQS-ES
Chloroflexus aurantiacus        --TVFTFSIH GAHNFPFRK- --QQSDLDIA LPDATGDTAY LDAL------ --EWGCA-RS
Dechloromonas aromatica         --SIFTFSIH GARNFPFDK- --EQSDLDIE LPDGCSDDAY LAHLE-EG-- --LGIAF-DA
Chromobacterium violaceum       --RIFTFSMH GARNFPFRR- --VDSDWDID LPDGTEDAAY LDALA-RALP ELFARAR---
Rubrobacter xylanophilus        --SVFTFSVH GEKNYPFRK- --GRSDLDVG LPDGADDGAF LEALS-EGLE RVLEGFR---
Thermus thermophilus            --SVFTLSLH GERNYPLKK- --ERSDLDVG LPDGTGDEAY LWALE-EALE KARAFR----
Deinococcus radiodurans         --TAFTLSIH GERNYPFRK- --ERSSLDLG LGDGVTDAEY LAVLTGQALP ALDAYR----
Pseudomonas putida              --EAITVSLH CEQNFPARK- --AQSDWDIP LPRGMGDTAY LKVVDDALNY LLPLYQ----
Pseudomonas fluorescens         --EAITVSLH CEKNFPARK- --AESDWDIP LPNGMGDADY LKVVDDALNY LLPLYQ----
Pseudomonas syringae            --DAITVSLH CEKNFPARK- --AQSDWDIP LPMGMGDADY LNVVDDLLNY LLPLYK----
Azotobacter vinelandii          --EAITVSLH CEQNFPARK- --ARSDWDIG LPRGMGDADY LKVVDDALNY LLPLYQ----
Phaeodactylum tricornutum       HSKLYTLSLH CASNYPRLK- --AHSTWDVG LPDRMEDDEY MDALVRAVDV ALAAAQ----
Thalassiosira pseudonana        CNKLYTLDLH AESNYPHPK- --EKCTYDVP LPDDCDDELY LSSLGDALDR ALEEVN----
Ostreococcus tauri              -HRCYTFSAH AASNFPARK- --ARSTRDVE LPRSMNDDEY MSVVSSALRE SLEDFR----
Chlamydomonas reinhardtii       -D-VFTLSVH AASNFPARK- --QRSTLDIA LPDGTPDAVY LARVAEILPA VLRDFK----
Nostoc sp                       --SVFTFSMH CEINFPGTK- --QHSDLDVP LPVGMEDDAY LQTLASYLPD LLSEIK----
Anabaena variabilis             --SVFTFSMH CEVNFPGTK- --QHSDLDVP LPVGMEDDAY LQTLASYLPD LLSEIK----
Nostoc punctiforme              --SVFTFSMH CEVNFPGTK- --QNSDLDVP LPVGMEDDAY LQTLANYLSD LLSKVK----
Trichodesmium  erythraeum       --TVFTFSMH CGINFPSRK- --QVSDLDVP LPEGMDDELY LQTLAQHLPY LLSEVK----
Crocosphaera watsonii           --TVFTFSMH CESNFPREK- --QKSDLDVA LPKGLDDDGY LQILSQYLPD LLSEIK----
Synechococcus sp                --TVFTFSMH CEVNFPSQK- --QRSDLDLG LPEGLDDDGY LQILAHHLED LLSQVK----
Synechococcus elongatus         --RVFTFSMH CEANFPARK- --QHSDCDVP LPEGMEDDAY LQQLAQYLPD LLSQVR----
Gloeobacter violaceus           --GVFTFSMH CEQNFPGRK- --QRSDLDVP LPIGLDDDTY LRVLNEHLPG LIETVR----
Nematostella vectensis          --NVFTFSAH SEKNFPLRK- --QTSNLDLS LECGMDDLEY LTTVCAHLTW LLDMWR----
Locusta migratoria              ---------- ---------- ---------- ---------- ---------- ----------
Callinectes sapidus             --DVFTLSFH CQNNFPLRK- --QKSDLDVG LAVGTDDKQY LNTLAEYLPD IMDSFR----
Platynereis dumerilii           --SIFTFSMH CQSNFPFVK- --QNSDLDVG LADHIGDAEY MRELQTHLPV ILETFH----
Strongylocentrotus purp.        --SVFTLSVH CGKNYPLKK- --QQSDLDVS VDRGTGDDDY MRIIQDHIPS VLTNFR----
Takifugu rubripes               --RVFTFSVH CGKNFPLRK- --QQSDLDVS VDDGLEDKDY LSTVEDHLPW LLDTFR----
Oryzias latipes                 --AVFTFSVH CGKNFPLRK- --QESDLDIS LEDGLEDEEY LSTVEAYLPW LLQTFR----
Gasterosteus aculeatus          --DVFTFSVH CXKNFPVRK- --QQSDLDVS VKDGLEDKEY LSTVEAHLPW LLETFR----
Pimephales promelas             --NVFTFSVH CGKNFPVRK- --QQSDLDVS LEDGTEDKEY LSKVQEHLPW LLETFQ----
Magnetospirillum magn.          --DLFTLSIH CERNYPYDK- --VPGDLDIG LPDGLGDADY LAVLEAHLPA LVQGFD----
Mesorhizobium loti              --GVFTFSMH GERNYPVRK- --IASDLDIA LPDGTGDAAY LRRLATILPE LSARAR----
Caulobacter crescentus          --SVFTFSMH AEKNFPHRK- --ASSDLDIE LSDGTDDGAY LAKLEAILPA LLISVR----
Erythrobacter litoralis         --DIVTFSMH AEKNFPVRK- --ARSNHDVA LPDGMEDVAY LETLAEHLPR LLDDVA----
Emiliania huxleyi               --RAATFSMH AARAFPVRK- --QRSTVDVG LPNGTGDEEY LRLLREHLPP LLDEHR----
Vibrio vulnificus               --DIVTLSFH CDKNFPARK- --PDSDLDIG LPRECGDEAF LAAFKEVVPM AIRLHQ----
Vibrio parahaemolyticus         --DIVTLSFH CDKNFPARK- --PQSDLDVP LAKGTDDETF LMTFKEVVEM ALNLHR----
Vibrio cholerae                 --EIITLSFH CDKNFPARK- --PASSMDVG YANQTGDEEF LSTFIQVVEM AVNLHR----
Shewanella oneidensis           --GIISCSIH CKDNFPSRK- --QHSHYDIE LVKGTDDSAY LDTVEQTLEL LIRLHQ----
Idiomarina loihiensis           --RVFTCSIH GERNFPFSK- --ASSNLDIP LAKHTSDKEY LAALENTLER INREIK----
Solibacter usitatus             --SVFTLSIH QFNNYPSEK- --PLSSLDIH LTDGIGDAEY LHRLGNGYRA ALTMFK----
Schmidtea mediterranea          --EVFTFSMH GAKNYPLFK- --EVSNLDIE LPDKTSDVEF LETLEQAV-P RIFLHD----
Drosophila melanogaster         ---VYIFDMY NAFVYPRDHV AKESIRCAVE LRNYTEDGFY LRQLKRCLMQ SLAEF-----
Chlamydomonas reinhardtii       ---LYILDMY NAGVFPRD-- ---------- ------D--- --EAK----- ----------
Anopheles gambiae               ---VFILDMY NYRIYPRDQH AKLAIRRAVE LKPHTDDEEY LRKLKHCLSQ SIAEF-----
Arabidopsis thaliana            ---VYILDMY NPEIYPFDYR ARRFIDQKVE VMSGTTTDEY LRKLDEALEV ASRNF-----
Aquilegia formosa               ---VYILDVY NPEIYPHDLE ARNYIDQKVE VVTGTTTDVY LERLDKALKV AGSMF-----
Oriza sativa                    ---VYTLDMY NAGIYPYDHV AKRYIDQKVE LVSGTKTEDY LDQLDKALKV AESRF-----
Hordeum vulgare                 ---VYILDMY NAGIYPFDYA AKRYIDQKIE LVSGTETDDY LDQLDKALKV AQTRF-----
Picea engelmannii               ---VYILDMY NSEIYPLDYA ARTSIQQRVE LLSGASTREY LERLDKELEV AQEAF-----
Homo sapiens                    ---VYIMDVY NRHIYPGDRF AKQAIRRKVE LEWGTEDDEY LDKVERNIKK SLQEH-----
Mus musculus                    ---VYIMDVY NRHIYPGDRF AKEAIRRKVE LEWGTEDEEY LEKVERNVRR SLQEH-----
Gallus gallus                   ---VYIMDAY NRYIYPGDGF AKRAIKRKVE LEWGTEDTEY LQKVHTHVEG ALNEL-----
Danio rerio                     ---VYIMDVY NRHIYPGDGY AKRAIKRKVE LDWGTEDSEY LQKVDLHSEG ALNEA-----
Strongylocentrotus purp.        ---VYILDVY NRHIYPRDGF AK-------- ---------- ------NLEA ALNEF-----
Tribolium castaneum             ---VYIIDVY NKGIYPFDKL AKEYITRKVE LAHFTEDDEY LDKVAKNLTE ALAEF-----
Locusta migratoria              ---VYILDIY NKGIYPNDGE AKKAIRRAVQ LEHFTADAEY LDLVEVNLEE ALREF-----
Ciona intestinalis              ---VFVMDVY NRNIYPHDGY AKRGIKRKVE LTSGVDDQTY LPLVKSHLTE SIGEF-----
Dugesia ryukyuensis             ---VFILDIY NEGIYPHDEE AKRGIDISVG LMSYTKDKEY LEKLNENLDK AFDQF-----
Solanum tuberosum               ---VFILDMY NPGIYPLDFE ARRYIDLGVE VRVGLRQMNT ---------- ----------
Antirrhinum majus               ---------- ---------- ---------- ---------- ---------- ----------
Triticum aestivum               ---GYILDMY NAWI------ ---------- ---------- ---------- ----------
Zea mays                        ---VYILDMY NAGIYP-FFT AKQYIDQK-- ---------- ---------- ----------
Heterodera glycines             ---VYILDLF NASIYPGDSD ALKSVSRAVP LPFGAEDDFY LRRLEMELDA A---------
Caenorhabditis briggsae         ---VFVFDVF NPYVYPHDRE ARQFINKAVH VNSHTTDTSY ISELRKQLTQ CLENRQNLTP
Caenorhabditis elegans          ---VFVFDVF NPYVYPHDRE ARQFINRAVH VNGHTTDTSY LSELRKQLAQ CLIDREKTTP
Tetrahymena thermophila         ---TFILDFY NHSIYPADTF AAKGISLSKN VDFDTSDSEY ISMLRKTLQK ILDQF-----
Paramecium tetraurelia          ---VYIIDFY NSYIYPGDHI AEQAISCFEH IDKDTSDQQY IKTLQRDLET HLKD------
Leptospira interrogans          --DVFTFSMH QENLYP-KK- --EKSDLDIS LEEGIGDKEY LELLEKSLRK IESDFK----
```

```
                                421
Cytophaga hutchinsonii          LQPDHIFFQS GVDVLETDKL GKLSLSREGC KNRDRMVLDR CKK-Y-KIPV TISLGGGYSE
Nematostella vectensis          FCPTFIFFQS GVDVLETDKL GRLGLSIEGC KKRDQFVFKK VKE-L-GVPV VCSMGGGYSP
Psychrobacter cryohalolentis    VAPDMIFYQS AVDVLATDKL GKLGLTIEGC KARDEYVLRQ AKA-A-KIPI AIVMGGGYSE
Phaeodactylum tricornutum       EPFDLVFFQA GVDVLAQDRL GRMSLTPVGV QRRNQLVYEF CAS-Q-SLPL VICMGGGYGD
Thalassiosira pseudonana        KPFDLIFYQA GVDIHEDDRL GRLSITSEGM SRRNSLVYDF ADR-M-RAPL VITMGGGY--
Cyanidioschyzon merolae         HDPDLVFYQA GVDALAEDKL GRLALTRAGL RLRNQIVLDY ILQES-EAGI VICMGGGYAD
Ostreococcus tauri              YEPGLVFYQA GVDALAQDSF GRLGMTRNGL LRRNNAVYSM CIS-T-NTPL VITMGGGYSK
Ralstonia metallidurans         D-PDLLIYLA GADPHEGDRL GRLKLTMAGL AQRDSMVFEA ARSR--GLPV AVAMAGGYGN
Ralstonia solanacearum          A-PRLIIYLA GADPHEGDRL GRLRLTMDGL ARRDRQVFDF AFLR--RIPI AVTMAGGYGN
Rubrivivax gelatinosus          P-PGLVFYLA GADPHEGDRL GRLRLSAAGL AERDRRVFAA CAER--GIPV VMTMGGGYGV
Polaromonas sp                  --PGLVVFLA GADPFEGDRL GRLALSFDGL EARDRRVFDW AWQR--RIPL TFSMAGGYGV
Pirellula sp                    GQFDLVIYLA GADPYKNDRL GRLSLTMDGL RRRDELVLQW CHHN--DLPV AIAMAGGYSV
Rhodopirellula baltica          GQFDLVIYLA GADPYKNDRL GRLSLTMDGL RRRDELVLQW CHHN--DLPV AIAMAGGYSV
Chloroflexus aurantiacus        LQLPALIW-- ---------- ---------- ---------- ---------- ----------
Dechloromonas aromatica         SRPDLVIYLA GADPYHDDRL GRLGLSFAGL AERDRLVLAR CKAN--RIPV AIAMAGGYAR
Chromobacterium violaceum       --PDLVCYLA GADPYHGDRL GRLALSKQGL AERDRMVMEA CRRY--DAAL AVTMAGGYSV
Rubrobacter xylanophilus        --PQLAVYLA GADPFAGDRL GRLAVSKEGL AERDRIVLEG CRGR--GVPV AVTMAGGYAR
Thermus thermophilus            --PDLVFYNA GVDVLKGDRF GRLALSPEGV RRRDERVFRF VKAL--GAPL VVVMGGGYNR
Deinococcus radiodurans         --PDLLLYLA GADVLAGDRF GRFALTLSGV SARNRAVFRW AQAA--GVPI VTMMAGGYNS
Pseudomonas putida              --PDLVLYDA GVDVHKDDAL GYLQLTDAGV AARDEAVLRH CLGR--DIPV MGVIGGGYSK
Pseudomonas fluorescens         --PDLVLWXA GVXVLKXXAL GYLPLXXXGV LEGDXSVMRX XLX-----PS ----------
Pseudomonas syringae            --PDLVLYDA GVDVHKDDAL GYLQLTDQGL ANRDEAVLRH CLSR--DIPV MGVIGGGYSK
Azotobacter vinelandii          --PDLVLYDA GVDVHQDDAL GYLALSDAGL AARDGAVLRH CLAR--GIAV LGVIGGGYDR
Phaeodactylum tricornutum       --PDLVLYDA GVDVYRKDKL GRLHLTLDGI RRRDRWVLDR CVSA--RIPV AAVVGGGY--
Thalassiosira pseudonana        --PQLVLYNA GVDVYHSDKL GRLSLSWEGM KQRDIHVVRR CLID--NIPV ACVVGGGY--
Ostreococcus tauri              --PELVIYDA GVDVTANDAL GHLDLTFEGL YRRERMVLDT CLGS--GIPV AGVVGGGYSP
Chlamydomonas reinhardtii       --PDLVLYDA GVDPHVDDAL GRLALTDAGL ARRERLVLDT CLGW--GVPV AGYVGGG---
Nostoc sp                       --PDLVLYDA GVDPHIGDRL GKLALTDTGL FRREMQVLTT CVSA--GYPV ACVIGGGYAD
Anabaena variabilis             --PDLVFYDA GVDPHIGDRL GKLALSDTGL FRREMQVLTT CISA--GYPV ACVIGGGYAD
Nostoc punctiforme              --PDLVFYDA GVDPHIGDRL GKLALTDAGI FRREMQVLST CMSS--GYPV ACVIGGGYAD
Trichodesmium  erythraeum       --PDLVLYDA GVDTHIHDAL GKLALTDTGI FRREMQVLST CLGK--GYPV ASVIGGGYTK
Crocosphaera watsonii           --PDLILYDA GVDIHANDHF GKLSLTDTGI YRREMLVLSS CIAE--GYPV ASVIGGGYAK
Synechococcus sp                --PDLVFYDA GVDTHVGDRL GKLAMTNTGL YRRERLVLST CLAA--GYPV ACVIGGGYAK
Synechococcus elongatus         --PNLVIYNA GVDPHGGDRL GKLALTDTGL FRREMQVLET CVRQ--GYPV AAVLGGGYCE
Gloeobacter violaceus           --PDLVLYDA GVDPHRSDKL GKLALTDRGL FERDRAVLGL CLKR--GIPV AAVIGGGYDN
Nematostella vectensis          --PDIVLYDA GVDPHVDDVL GRLKLTDMG- ---------- ---------- ----------
Locusta migratoria              ---------- ---------- ---------- ---------- ---------- ----------
Callinectes sapidus             --PDIILYDA GVDPHEKDEL G--------- ---------- ---------- ----------
Platynereis dumerilii           --PDLILYDA GVDPHVKDEL GKLDLTDQGL FDRDYYVLDE GIRR--GIPL VTVIGGGYSH
Strongylocentrotus purp.        --PGLVFFDA GVDPHKDDAL GYLELTDQGL FRRDYWVMNE VIQR--GIPC VTVIGGGYDK
Takifugu rubripes               --PDLVLYDA GVDPHREDEL GKLCLTDQGL YQRDLYVMQT VVGK--GVPV ATVIGGGYSR
Oryzias latipes                 --PDLVLYDA GVDPHWEDEL GRLHLTDQGL YRRDLFVMKT TVNQ--GVPI ATVIGGGYSR
Gasterosteus aculeatus          --PRLVLYDA GVDPHRDDEL GPLRLTDRGL YQRDLYVIKA W--------- ----------
Pimephales promelas             --PDLVLYDS GVDPHWEDEL GRLRLTDEGL YQRDLYVLQT VIKK--GIPV ATVIGGGYSR
Magnetospirillum magn.          --PDLIFYNA GVDSHRDDRL GRLRLSDAGL RARDRYVVGL ARRR--NIPL CTVIGGGYGS
Mesorhizobium loti              --WDIVFYNA GVDVHAEDRL GRLALSNGGL RARDEMVIGH FRAL--GIPV CGVIGGGYST
Caulobacter crescentus          --PDIVFFNA GVDPHADDKL GRLALTDEGL ARREAYVLGA CLSS--EIPV VGVIGGGYDA
Erythrobacter litoralis         --PDIVLYQA GVDPHEDDKL GRLNLTSEGL DERDRFVVRE VRRR--GLPI ASALGGGYGE
Emiliania huxleyi               --PDLVLYDA GADAVRTFCR GHLSLTDAGL RKRDFFVMDE CLSR--GVPV ATVIGGGY--
Vibrio vulnificus               --PDLIIYDA GVDIHHEDEL GYLNVSTDAL YQRDAFLFGQ AKAN--AIPV AAVVGGGYRT
Vibrio parahaemolyticus         --PDMVIYDA GVDIHQDDEL GYFDVSTQAI FERDRFLFQL MKNR--GIPV AAVVGGGYRT
Vibrio cholerae                 --PDLILYDA GVDIHNDDEL GYLSISQAAI AQRDRFMLGL AKQE--SIPI ACVIGGGYRE
Shewanella oneidensis           --PDLILYDA GVDIHQDDDL GHLQISQQGL YQRDVTVLSM ARAA--NIPV AAVIGGGYSR
Idiomarina loihiensis           --PDLILYDA GVDIYQRDEL GHLNISLAGI FQRDLTVLRF AKRH--KTPL AAVIGGGYQR
Solibacter usitatus             --PELLMYVA GADPYMEDQL GGLSLTFEGL MERDRLVIRT ALTH--SVPV AIVLAGGYAQ
Schmidtea mediterranea          --PDIM---- ---------- ---------- ---------- ---------- ----------
Drosophila melanogaster         -RPDMVVYNA GTDVLEGDPL GNLAISAEGV IERDRLVFST FRALG--IPV VMLLSGGYLK
Chlamydomonas reinhardtii       -----VVYNA GTDVLAGDPL GRLGVSHAGV VERDELVG-- ARARG--VPI AMLLSGGYAK
Anopheles gambiae               -EPNFIIYNA GTDILKGDPL GLLDITPEGV VERDEFVFRS ALERS--IPL VMLLSGGYLR
Arabidopsis thaliana            -QPELVIYNA GTDILDGDPL GLLKISPDGI TSRDEKVFRF AREKN--IPL VMLTSGGYMK
Aquilegia formosa               -DPELVVYNA GTDILDGDPL GGLKVSPDGV ISRDEKVFRF AKDKK--IPL VMVTSGGYMM
Oriza sativa                    -QPQLIVYNA GTDILDGDPL GRLKISPQGV VIRDEKVFRF AKDQS--IPL LMLTSGGYMK
Hordeum vulgare                 -QPQLIIYNA GTDILDGDPL GNLKISPEGV VIRDEKVFKF ---------- ----------
Picea engelmannii               -DPELIIYNA GTDILNGDPL GRLKVDPDGV IKRDEKVFAF AQQKK--VPI VMLTSGGYMK
Homo sapiens                    -LPDVVVYNA GTDILEGDRL GGLSISPAGI VKRDELVFRM VRGRR--VPI LMVTSGGYQK
Mus musculus                    -LPDVVVYNA GTDVLEGDRL GGLSISPAGI VKRDEVVFRV VRAHD--IPI LMVTSGGYQK
Gallus gallus                   -KPDIIVYNA GTDILDGDPL GGLAISPQGI VKRDEVVFKA ARSRG--IPI LMVTSGGYQK
Danio rerio                     -RPDIIIYNA GTDILDGDPL GGLAISPQGI IKRDEIIFRA ARRRG--IPI LMVTSGGYQK
Strongylocentrotus purp.        -VPDILVYNA GTDSLEGDPL GALSISLQGI IKRDMMVFEF ARDRPKPIPI VMVTSGGYQR
Tribolium castaneum             -CPQLIVYNA GTDILKGDAL GCLSVSPQGI IERDELVFRE ARSRN--IPI VMLTSGGYLK
Locusta migratoria              -HPDIIVYNA GTDI------ ---------- ---------- ---------- ----------
Ciona intestinalis              -NPQLIVYNA GTDILQGDQL GRLNIS--GI VKRDEIVFAL AKGRG--IPI VMLTSGGYQR
Dugesia ryukyuensis             -EPQLIVYNA GTDCLLGDPL GRLSISEKGI VDRDEIVFKK AKERK--CPI VMLTSGGYQH
Solanum tuberosum               ---------- ---------- ---------- ---------- ---------- ----------
Antirrhinum majus               ---------- ---------- ---------- ---------- ---------- ----------
Triticum aestivum               ---------- ---------- ---------- ---------- ---------- ----------
Zea mays                        ---------- ---------- ---------- ---------- ---------- ----------
Heterodera glycines             ---------- ---------- ---------- ---------- ---------- ----------
Caenorhabditis briggsae         PGFDFILYNA GTDCLFGDPL GAMNLSPQCI ISRDEVVFKL AREKG--IPI CMLTSGGYQK
Caenorhabditis elegans          PGFDFIMFNA GTDCLLGDPL GAMKLSPQCI IARDEVVFNL AKSKG--IPI CMVTSGGYQK
Tetrahymena thermophila         -KPEFLLYNA GTDCLEGDRL GQMNLSQNCI IQRDQVVFEE CLNRD--IPL TMVLSGGYQQ
Paramecium tetraurelia          -DMEFLIYNA GTDIMAGDPL GHCCISAAGI KRRDEVVFKW AQHKK--IPF LMLLSGGYQK
Leptospira interrogans          --PDLIFYIA GADPFEGDSL GDLKLTFQGL RKRDQIVRDF AYSLN-DTRV VILPAGGYAK
```

```
                              481
Cytophaga hutchinsonii        KIIDIVEAHC NTFRLAQELY F--------- ---------- ---------- ----------
Nematostella vectensis        DIRQII---- ---------- ---------- ---------- ---------- ----------
Psychrobacter cryohalolentis  DIEDVVEAHC NTFRLAQQIF FDEITE---- ---------- ---------- ----------
Phaeodactylum tricornutum     PLESTCRQ-- ---------- ---------- ---------- ---------- ----------
Thalassiosira pseudonana      ---------- ---------- ---------- ---------- ---------- ----------
Cyanidioschyzon merolae       PITKSVDAHA DVY------- ---------- ---------- ---------- ----------
Ostreococcus tauri            PIQASLDAHV DVFRSA---- ---------- ---------- ---------- ----------
Ralstonia metallidurans       QIEDTVAVHT QTIMLAARYH AQLAAERASV S--------- ---------- ----------
Ralstonia solanacearum        NIDDTVAVHA QTIALAALHA ARWAAGEAAT SVSSSSLPLS A--------- ----------
Rubrivivax gelatinosus        NIDDTVTVQL ASYREALAGW SAWRARAVVT ADGTAAAGLR ---------- ----------
Polaromonas sp                NIDETVQVQM NTYRVALEYW AKWNP----- ---------- ---------- ----------
Pirellula sp                  EVKEIVDIHS QTLHIAKVWS LSR------- ---------- ---------- ----------
Rhodopirellula baltica        EVKEIVDIHS QTLHIAKVWS LSR------- ---------- ---------- ----------
Chloroflexus aurantiacus      ---------- ---------- ---------- ---------- ---------- ----------
Dechloromonas aromatica       QINDTVSIHS TTIHLAKALL GS-------- ---------- ---------- ----------
Chromobacterium violaceum     PITDTVTIQT ETVRLACEIF GSSPTNH--- ---------- ---------- ----------
Rubrobacter xylanophilus      DISDTVEIHL NSVLRAAALH AAGSG----- ---------- ---------- ----------
Thermus thermophilus          DPRLTVEAHA ATYRLALSSL A--------- ---------- ---------- ----------
Deinococcus radiodurans       DHALTVEAHA SVVLDGLDVL G--------- ---------- ---------- ----------
Pseudomonas putida            DRTALARRHG ILHHSAARVI GCSQ------ ---------- ---------- ----------
Pseudomonas fluorescens       ---------- ---------- ---------- ---------- ---------- ----------
Pseudomonas syringae          DRLALARRHG ILHHSAQRVW NDMGL----- ---------- ---------- ----------
Azotobacter vinelandii        DRAALARRHG ILHHGAARLW RELGLG---- ---------- ---------- ----------
Phaeodactylum tricornutum     ---------- ---------- ---------- ---------- ---------- ----------
Thalassiosira pseudonana      ---------- ---------- ---------- ---------- ---------- ----------
Ostreococcus tauri            DLDEIANRHA VLHRVAQEMF TDHGL----- ---------- ---------- ----------
Chlamydomonas reinhardtii     ---------- ---------- ---------- ---------- ---------- ----------
Nostoc sp                     DMTSLVWRHS LVHRAASQVY RQYKL----- ---------- ---------- ----------
Anabaena variabilis           DMTSLVWRHS LLHRAASEVY RQYKL----- ---------- ---------- ----------
Nostoc punctiforme            DMKSLVWRHS LLHRAASEVY QQYKL----- ---------- ---------- ----------
Trichodesmium  erythraeum     DMNSLVYTHS LLHRAARDVF YQYRL----- ---------- ---------- ----------
Crocosphaera watsonii         DLKSLVYRHS LLHRAAKDVY KFYQL----- ---------- ---------- ----------
Synechococcus sp              NI------HD LYIAIPCSIG QRGMFS---- ---------- ---------- ----------
Synechococcus elongatus       DFNALVYRHS LCQRAASDVY RTWL------ ---------- ---------- ----------
Gloeobacter violaceus         NLDALVARHA LLHRAAAEVY RRA------- ---------- ---------- ----------
Nematostella vectensis        ---------- ---------- ---------- ---------- ---------- ----------
Locusta migratoria            ---------- ---------- ---------- ---------- ---------- ----------
Callinectes sapidus           ---------- ---------- ---------- ---------- ---------- ----------
Platynereis dumerilii         DLDELSIRHT IVHRAATKIW NEYNL----- ---------- ---------- ----------
Strongylocentrotus purp.      DIDRLAARHS IVHRAAKKAL LACKKAITAT VCNVNGSNSL LVGSWNPFGK KGDYDVKSVL
Takifugu rubripes             DIDKLAIRHS IVHRAATQV- ---------- ---------- ---------- ----------
Oryzias latipes               DMDRLALRHS IVHRAATQVW RECGM----- ---------- ---------- ----------
Gasterosteus aculeatus        ---------- ---------- ---------- ---------- ---------- ----------
Pimephales promelas           DIDQLARRHS IIHRAASKVW SEYGL----- ---------- ---------- ----------
Magnetospirillum magn.        D--------- ---------- ---------- ---------- ---------- ----------
Mesorhizobium loti            DVPALASRHA ILFEVASTYA ---------- ---------- ---------- ----------
Caulobacter crescentus        DIDRLAARHA ILHRTAKSLC SL-------- ---------- ---------- ----------
Erythrobacter litoralis       DQREVGARHA RSMLAMAAEN RKHPRPQTAR ARSQSQETAS ---------- ----------
Emiliania huxleyi             ---------- ---------- ---------- ---------- ---------- ----------
Vibrio vulnificus             DHEALVPLHL QLLNAAFDVY RSTPD----- ---------- ---------- ----------
Vibrio parahaemolyticus       NHADLVPIHM QLIKAATKVF AS-------- ---------- ---------- ----------
Vibrio cholerae               DHTALVPLHL ELLKAALSAG Y--------- ---------- ---------- ----------
Shewanella oneidensis         DALQLSQRHS QLFIAANHLW LQTQ------ ---------- ---------- ----------
Idiomarina loihiensis         NLPRLVRAHA QLLRASYYVY TENKLT---- ---------- ---------- ----------
Solibacter usitatus           SVEDTITIHA NTAAVAKDVL GKVRL----- ---------- ---------- ----------
Schmidtea mediterranea        ---------- ---------- ---------- ---------- ---------- ----------
Drosophila melanogaster       ASAGVITDSI VNLRL----Q GLLN------ ---------- ---------- ----------
Chlamydomonas reinhardtii     DSAAVITASL TNL------- ---------- ---------- ---------- ----------
Anopheles gambiae             SSARVIANSI VNLRD----K ALLPTVQ--- ---------- ---------- ----------
Arabidopsis thaliana          SSARVIADSI ENLSR----Q GLIQTRPE-- ---------- ---------- ----------
Aquilegia formosa             SSARVIADSI VNLRK----K HLINLEIPK- ---------- ---------- ----------
Oriza sativa                  SSARVIADSI INLSN----K NLIELGSQLG ---------- ---------- ----------
Hordeum vulgare               ---------- ---------- ---------- ---------- ---------- ----------
Picea engelmannii             SSARVIANSI INLSN----K HLIELNK--- ---------- ---------- ----------
Homo sapiens                  RTARIIADSI LNLFG----L GLIGPESPSV SAQN------ -----SDTPL LPPAVP----
Mus musculus                  RTARIIADSI LNLHD----L GLIGPEFPCV SAQN------ -----SGIPL LSCAVP----
Gallus gallus                 RTARIIADSI LNLHN----L GLIDKELATS EAESPKVDQM IRDSVTDLTN LTCSDG----
Danio rerio                   KTARIIADSI LNLHR----Q GLIGEEALDV AGPSHVSLMM SKSVSSGATL AAI-------
Strongylocentrotus purp.      NNADIIAASI LNLWR----R GLI------- ---------- ---------- ----------
Tribolium castaneum           KSAKIIATSI KNLHD----S GLITGPQ--- ---------- ---------- ----------
Locusta migratoria            ---------- ---------- ---------- ---------- ---------- ----------
Ciona intestinalis            TTARIIANSI LSLRN----N GLI------- ---------- ---------- ----------
Dugesia ryukyuensis           TNAAIIANSI WESF------ ---------- ---------- ---------- ----------
Solanum tuberosum             ---------- ---------- ---------- ---------- ---------- ----------
Antirrhinum majus             ---------- ---------- ---------- ---------- ---------- ----------
Triticum aestivum             ---------- ---------- ---------- ---------- ---------- ----------
Zea mays                      ---------- ---------- ---------- ---------- ---------- ----------
Heterodera glycines           ---------- ---------- ---------- ---------- ---------- ----------
Caenorhabditis briggsae       NNAHLIAKSI ENLHS----K NLINLGIN-- ---------- ---------- ----------
Caenorhabditis elegans        DNALLIAKSI ENLQS----K NLISIK---- ---------- ---------- ----------
Tetrahymena thermophila       INAPVIADSI LNLDQNLKIK NYNRKKLQNP ---------- ---------- ----------
Paramecium tetraurelia        ENTFAIGESI LQLIT----- ---------- ---------- ---------- ----------
Leptospira interrogans        DFYDTVTIHY NTIKIFAAD- ---------- ---------- ---------- ----------
```

	541	
Cytophaga hutchinsonii	----------	--------
Nematostella vectensis	----------	--------
Psychrobacter cryohalolentis	----------	--------
Phaeodactylum tricornutum	----------	--------
Thalassiosira pseudonana	----------	--------
Cyanidioschyzon merolae	----------	--------
Ostreococcus tauri	----------	--------
Ralstonia metallidurans	----------	--------
Ralstonia solanacearum	----------	--------
Rubrivivax gelatinosus	----------	--------
Polaromonas sp	----------	--------
Pirellula sp	----------	--------
Rhodopirellula baltica	----------	--------
Chloroflexus aurantiacus	----------	--------
Dechloromonas aromatica	----------	--------
Chromobacterium violaceum	----------	--------
Rubrobacter xylanophilus	----------	--------
Thermus thermophilus	----------	--------
Deinococcus radiodurans	----------	--------
Pseudomonas putida	----------	--------
Pseudomonas fluorescens	----------	--------
Pseudomonas syringae	----------	--------
Azotobacter vinelandii	----------	--------
Phaeodactylum tricornutum	----------	--------
Thalassiosira pseudonana	----------	--------
Ostreococcus tauri	----------	--------
Chlamydomonas reinhardtii	----------	--------
Nostoc sp	----------	--------
Anabaena variabilis	----------	--------
Nostoc punctiforme	----------	--------
Trichodesmium erythraeum	----------	--------
Crocosphaera watsonii	----------	--------
Synechococcus sp	----------	--------
Synechococcus elongatus	----------	--------
Gloeobacter violaceus	----------	--------
Nematostella vectensis	----------	--------
Locusta migratoria	----------	--------
Callinectes sapidus	----------	--------
Platynereis dumerilii	----------	--------
Strongylocentrotus purp.	DSGTAIEVYQ	NNLSSANC
Takifugu rubripes	----------	--------
Oryzias latipes	----------	--------
Gasterosteus aculeatus	----------	--------
Pimephales promelas	----------	--------
Magnetospirillum magn.	----------	--------
Mesorhizobium loti	----------	--------
Caulobacter crescentus	----------	--------
Erythrobacter litoralis	----------	--------
Emiliania huxleyi	----------	--------
Vibrio vulnificus	----------	--------
Vibrio parahaemolyticus	----------	--------
Vibrio cholerae	----------	--------
Shewanella oneidensis	----------	--------
Idiomarina loihiensis	----------	--------
Solibacter usitatus	----------	--------
Schmidtea mediterranea	----------	--------
Drosophila melanogaster	----------	--------
Chlamydomonas reinhardtii	----------	--------
Anopheles gambiae	----------	--------
Arabidopsis thaliana	----------	--------
Aquilegia formosa	----------	--------
Oriza sativa	----------	--------
Hordeum vulgare	----------	--------
Picea engelmannii	----------	--------
Homo sapiens	----------	--------
Mus musculus	----------	--------
Gallus gallus	----------	--------
Danio rerio	----------	--------
Strongylocentrotus purp.	----------	--------
Tribolium castaneum	----------	--------
Locusta migratoria	----------	--------
Ciona intestinalis	----------	--------
Dugesia ryukyuensis	----------	--------
Solanum tuberosum	----------	--------
Antirrhinum majus	----------	--------
Triticum aestivum	----------	--------
Zea mays	----------	--------
Heterodera glycines	----------	--------
Caenorhabditis briggsae	----------	--------
Caenorhabditis elegans	----------	--------
Tetrahymena thermophila	----------	--------
Paramecium tetraurelia	----------	--------
Leptospira interrogans	----------	--------
